# Long-term impacts of co-designed sustainable park improvements on physical activity and other wellbeing behaviours: a 7-year natural experimental study in a deprived urban area

**DOI:** 10.1186/s12966-026-01918-9

**Published:** 2026-04-21

**Authors:** Jack S. Benton, Jamie Anderson, Atticus Morley, Junyan Ye, Ellie Barker, Timothy Wu, Vanessa G. Macintyre, James Rothwell, Matthew Dennis, David P. French

**Affiliations:** 1 Department of Geography, School of Education, Environment and Development, The University of Manchester, Arthur Lewis Building, Oxford Road, Manchester, UK; 2 Manchester Urban Institute, School of Environment, Education and Development, The University of Manchester, Manchester, UK; 3 School of Social Sciences, The University of Manchester, Manchester, UK; 4 Manchester Centre for Health Psychology, Division of Psychology & Mental Health, School of Health Sciences, The University of Manchester, Manchester, UK

**Keywords:** Physical activity, Wellbeing, Urban parks, Green space, Built environment, Nature-based solutions, Natural experiment, Intervention, Systematic observation, Intercept surveys.

## Abstract

**Background:**

Creating or improving urban parks is a promising intervention for promoting physical activity (PA). However, robust natural experimental evidence of effectiveness remains limited, particularly regarding long-term impacts. We previously showed that co-designed, sustainable urban park improvements in a deprived UK area increased walking and other wellbeing-related behaviours up to 15 months post-intervention. We now examine whether these effects were sustained five years post-intervention (seven years after baseline).

**Methods:**

Two intervention sites were matched to two comparison sites, with two additional nearby comparison sites included to assess wider neighbourhood trends. Outcomes were assessed using systematic observations at baseline (2018), 15 months post-intervention (2021), and five years post-intervention (2025). The primary outcome was change in the number of people walking; secondary outcomes included vigorous PA, sedentary behaviour, social interactions, and taking notice of the environment. Multilevel mixed-effects negative binomial regression models compared changes between intervention and comparison sites, adjusting for day of week, time of day, and precipitation. Additional analyses compared 15-month (2021) and five-year (2025) follow-ups to assess how intervention effects evolved over time. Intercept surveys assessed self-reported outdoor space use at baseline (2019; *n* = 217) and follow-up (2025; *n* = 232).

**Results:**

From baseline to five years post-intervention, walking at the intervention sites significantly increased by 70% relative to the comparison sites (IRR = 1.70, 95% CI 1.02–2.82). Significant increases were also observed for sedentary behaviour, social interactions, and taking notice of the environment. Comparison of the 15-month and five-year follow-ups indicated some attenuation of intervention effects over time. Observations at nearby comparison sites suggested that these effects were not attributable to wider neighbourhood trends. The largest increases in observed park use were among young people and non-white ethnic groups. Intercept surveys corroborated the observational findings, showing significantly greater increases in self-reported outdoor space use in the intervention area.

**Conclusions:**

Co-designed, sustainable urban park improvements can generate long-term increases in walking and other wellbeing-related behaviours in deprived urban areas, with effects persisting for at least five years. Urban green space interventions, which can target areas with the greatest need, therefore represent an effective long-term strategy for increasing PA.

**Study protocol:**

Study protocol published in Open Science Framework before the first follow-up data collection finished (https://osf.io/zqgcn). Date of registration: 18 August 2020.

**Supplementary Information:**

The online version contains supplementary material available at 10.1186/s12966-026-01918-9.

## Background

By 2050, nearly 70% of the world’s population is expected to live in urban areas [2]. As cities continue to grow, urban green spaces will play an increasingly important role in supporting population health and wellbeing. A substantial body of evidence demonstrates that urban green spaces are associated with a range of health and wellbeing benefits [3], while also providing economic, social, and environmental co-benefits [4]. One key pathway through which urban green spaces influence health and wellbeing is by promoting physical activity (PA) [5].

Urban parks are a particularly important type of green space for promoting PA. In densely populated urban areas, they provide accessible opportunities for outdoor activity. Parks are especially well suited to promoting walking, as they offer accessible and attractive environments with dedicated path infrastructure that supports both recreational and utilitarian walking [6]. It is therefore unsurprising that walking is one of the most common forms of PA undertaken in urban parks [7].

Urban parks may be particularly important in socioeconomically deprived areas, where access to other health-promoting resources is often limited [8]. However, park use is typically lower in socioeconomically deprived areas [9, 10], reflecting inequalities in the availability, accessibility, and quality of parks and other green spaces [11]. Given that more deprived populations are generally less physically active [12] and experience poorer health [13], creating new or improving existing urban parks is a promising population-level intervention to increase PA and reduce health inequalities [14].

A large number of cross-sectional studies report positive associations between park availability, accessibility, and quality and PA [15–17]. However, cross-sectional designs cannot establish causality and provide limited insight into how parks should be designed or improved to promote PA. Because researchers rarely control the implementation of park interventions, producing robust evidence typically relies on natural experiments i.e., real-world interventions occurring outside researchers’ control [18]. By comparing changes in outcomes over time between populations exposed and unexposed to an intervention, natural experimental studies can generate valuable practice-based evidence to inform policy and practice [19].

Despite this, there are relatively few natural experimental studies evaluating the impacts of urban park interventions on PA [3, 20, 21], and many existing studies exhibit weak designs and a high risk of bias [22]. Further, most studies have been conducted in Australia and the United States [23], which differ from European countries in ways likely to influence PA behaviours, including climate, urban form, population density, and patterns of park use [24]. This limits the generalisability of findings to European urban settings.

A further major limitation of the evidence base is its focus on short-term impacts. Most natural experimental studies of parks assess PA less than one year post-intervention [23]. Such evaluations may underestimate the true value of park interventions, which are typically permanent changes to the built environment and therefore have the potential to produce sustained behavioural change over time. Conversely, initial increases in park use may attenuate over time as novelty effects diminish or maintenance challenges emerge. The lack of long-term evidence means that these longer-term impacts – whether sustained, attenuated, or amplified – remain poorly understood.

Only a small number of natural experimental studies have evaluated park interventions up to two years post-intervention. In Melbourne, one study has shown that installing a new playscape increased park visitation and activity, particularly among children [25], while another found that substantial upgrades increased visitation and moderate-to-vigorous PA, whereas more modest improvements produced no detectable effects relative to a control park [26], suggesting that intervention scale may influence effectiveness. Similarly, a recent study in Belgium reported increases in vigorous PA and sedentary park use two years after park renewal, although effects varied by age group and no overall increase in walking was observed [27]. However, whether such effects are sustained beyond two years remains largely unknown.

In addition to PA, urban parks may support other behaviours important for wellbeing (hereafter referred to as ‘wellbeing behaviours’). Two such behaviours are social interaction and taking notice of the environment, both of which align with the Five Ways to Wellbeing [28], a set of evidence-based behaviours linked to improved wellbeing. Social interaction (“Connect”) involves engaging with others through conversation or shared activities, which parks can facilitate by providing informal and accessible spaces for meeting and spending time together [29]. Taking notice of the environment (“Take Notice”) refers to consciously attending to the immediate environment – for example observing natural features, appreciating the landscape, or photographing wildlife – which exposure to natural environments may encourage and can contribute to psychological restoration [30]. Despite the plausibility of these pathways, robust natural experimental evidence examining the effects of park interventions on these broader wellbeing behaviours remains limited [31]. A sole focus on PA may therefore underestimate the wider health and wellbeing benefits that park interventions can generate.

To address these evidence gaps, the present study capitalised on a rare opportunity to conduct a prospective natural experimental study of co-designed, sustainable park improvements in a highly deprived area of Manchester, UK. We have previously demonstrated positive effects of this intervention on walking and other wellbeing behaviours at 3 and 15 months post-intervention [32]. The present study examines whether these effects were sustained in the longer term.

### Aim and objectives

The aim of this natural experimental study was to prospectively evaluate the long-term impacts of co-designed, sustainable park improvements in a deprived urban area in the UK. Specifically, the study assessed observed changes in walking and other wellbeing behaviours five years post-intervention (2025), equivalent to seven years after baseline, relative to: (a) baseline (2018); and (b) 15-months post-intervention (2021).

The specific objectives were to compare observed changes at these timepoints in:

1. The total number of people walking (primary outcome);
2. The total number of people engaging in vigorous PA, sedentary behaviour, social interactions, and taking notice of the environment (secondary outcomes);
3. Activity at nearby comparison sites, to assess wider neighbourhood trends;
4. The demographic characteristics of park users (exploratory outcomes).

A fifth objective was to assess changes in self-reported outdoor space use using intercept surveys to triangulate with any observed changes in PA behaviour.

## Methods

This study is reported in accordance with the Strengthening the Reporting of Observational Studies in Epidemiology (STROBE) guidelines for observational studies (Additional file 1) [33].

### Study design

This was a controlled pre-post prospective natural experimental study, conducted in accordance with an a priori registered study protocol [1].

### Intervention

The intervention is described in accordance with the Template for Intervention Description and Replication (TIDieR) reporting guidelines (Additional file 2) [34].

The intervention was a 1.4-hectare linear park opened in May 2020 in West Gorton, which is a highly deprived area of Manchester, UK – a large post-industrial city. The park was located within the top 3% most deprived areas in England in 2019 [35]. The intervention was commissioned by Manchester City Council and delivered as part of the EU-funded GrowGreen project, which aimed to demonstrate innovative nature-based solutions.

**Fig. 1:**
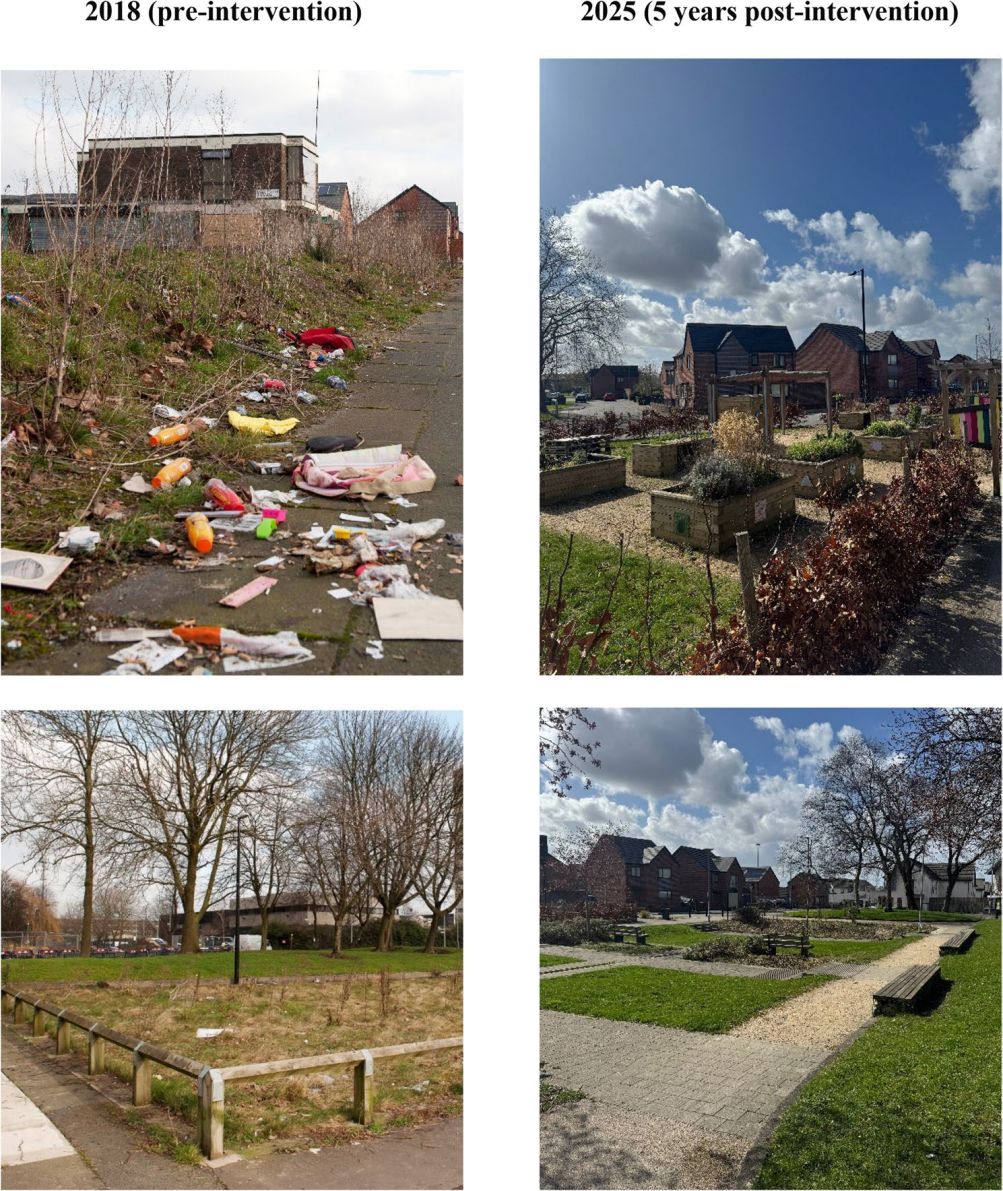
Intervention park at baseline in 2018 (left) and five-year post-intervention follow-up in 2025 (right). *Photographs taken by Jamie Anderson and Jack Benton*

Prior to the intervention, the space comprised a small park adjacent to brownfield land (Fig. 1). The pre-existing park had limited and outdated play facilities, low biodiversity, and was identified as being at risk of flooding.

The intervention introduced multiple sustainable design features, including rain gardens, biodiverse planting, new play facilities, and improved seating and sightlines. The park was organised into three distinct areas: (a) the Woodland, which involved upgrading the pre-existing park and provided substantially improved facilities for children and teenagers to play, socialise, and be physically active; (b) the Meadow Garden, previously brownfield land, which now includes new play features, a nature trail, picnic tables, and benches set within meadow planting; and (c) the Community Garden, designed to host community events and activities (e.g., gardening, children play sessions, cultural events) and featuring open lawns, paved areas, and a designated space for residents to grow vegetables and flowers. The estimated total cost of the intervention, including 25-year maintenance, was approximately £1.96 million (≈$2.37 million), excluding Value Added Tax.

The intervention was developed through an extensive co-design process led by delivery partners, involving local residents and community stakeholders across multiple stages of design. Engagement activities included information-sharing, consultation, and opportunities for residents to shape emerging proposals, and were conducted across three phases: baseline community engagement, concept design consultation, and sketch design consultation. Methods included youth engagement sessions, door-to-door questionnaires, drop-in sessions at accessible community locations (e.g., schools, medical centres, and shops), public consultation events, input from the Over-50s group and West Gorton Steering Group, and use of a mobile consultation unit to gather feedback on emerging designs. These activities engaged a wide range of local groups, including school pupils, healthcare staff, older adults, and other residents. Collectively, they supported participatory research and analysis by identifying local needs, priorities, and concerns, while also informing aspects of design projection by shaping key features of the final scheme.

Community input directly informed several key design decisions, including prioritising a single integrated park (rather than multiple smaller greening projects) to connect two neighbourhoods, enhancing recreational opportunities for children, providing seating and relaxation spaces for older adults, and incorporating community planting features. Residents also identified safety as a key concern, particularly due to restricted visibility caused by landscaped mounds, which was addressed in the redesign through improved sightlines across the park.

### Comparison group

#### Matched comparison sites

For the main analyses, two matched comparison sites in Greater Manchester were identified using a recently developed matching approach [36], based on fifteen key variables associated with PA (Additional file 3). First, neighbourhoods most similar to the intervention area were identified at the Lower Layer Super Output Area (LSOA) level using spatial data on population density, street connectivity, area-level deprivation, and greenness. Second, specific candidate sites within these neighbourhoods were identified through staged screening using Google Street View, followed by in-person site environmental audits and observations to assess footfall.

**Fig. 2:**
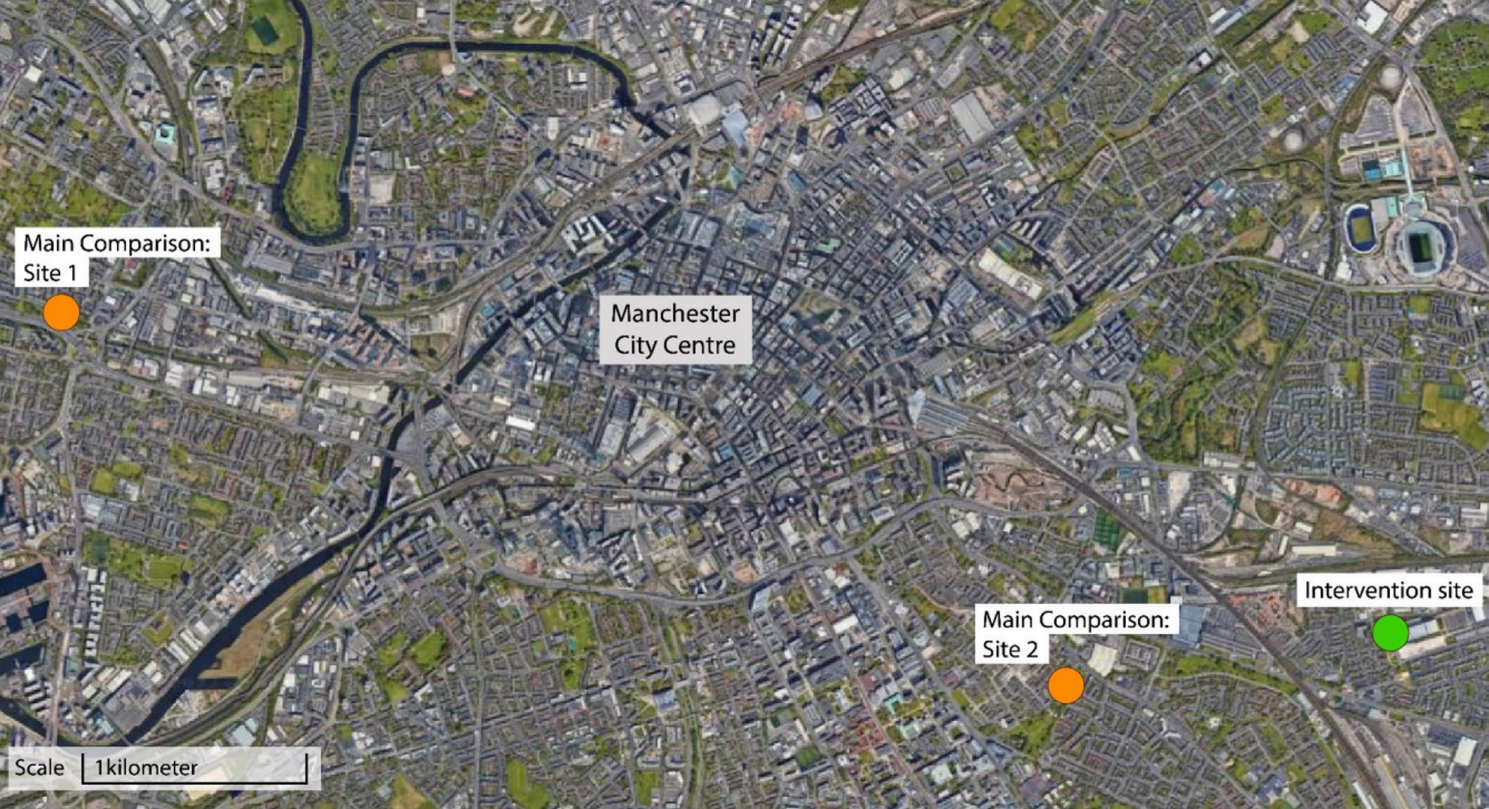
Map showing locations of the intervention and matched comparison sites. *Image originally published in Anderson et al.* [32]

The two matched comparison sites were located approximately 1.2 km and 5.5 km from the intervention park (straight-line distance) and were situated in separate neighbourhoods (Fig. 2), thereby reducing the likelihood of contamination between study sites. For analysis, the two comparison sites were pooled into a single comparison group to increase statistical power and reduce the influence of site-specific characteristics.

#### Nearby comparison sites

**Fig. 3:**
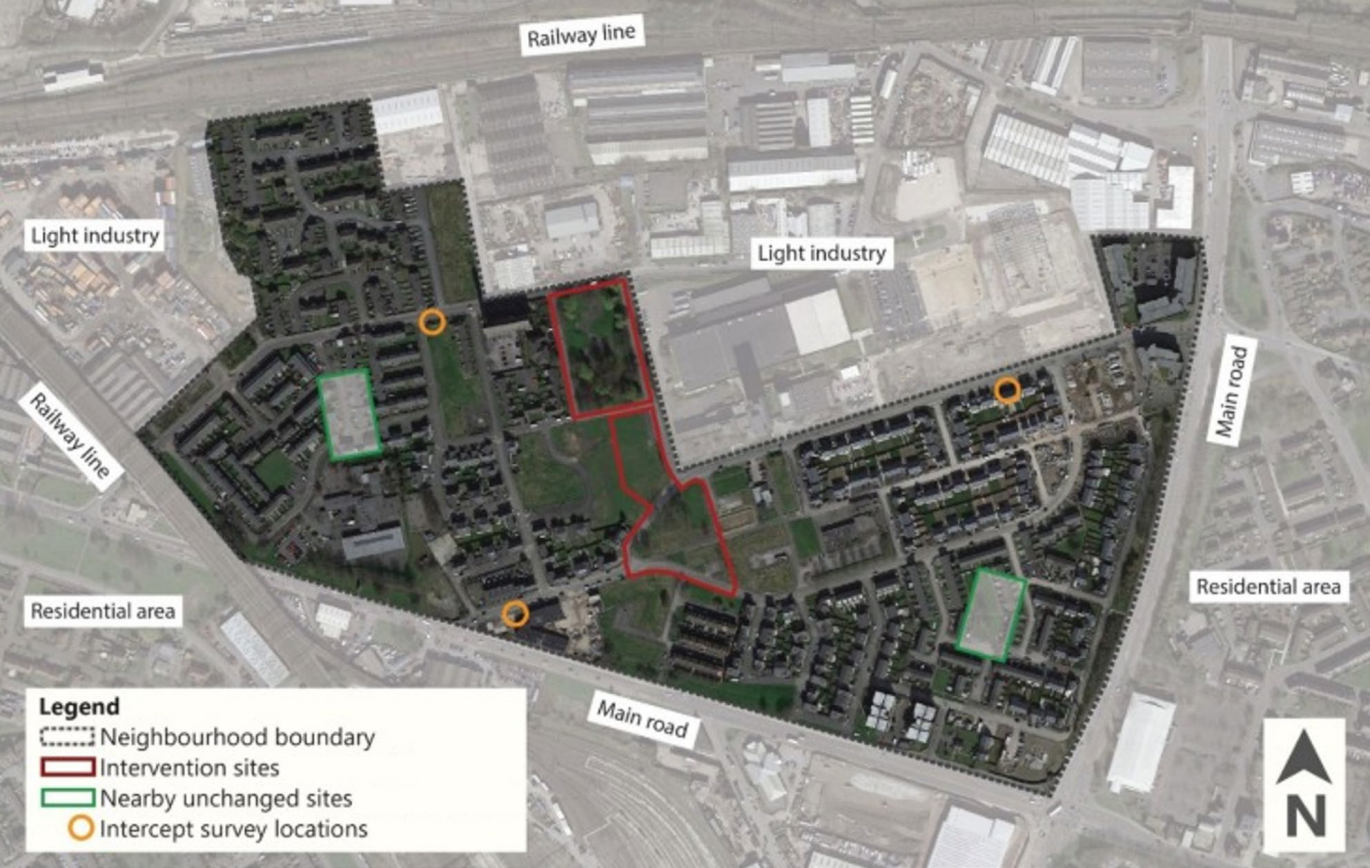
Map showing locations of all study sites in the intervention area. *Image originally published in Anderson et al.* [32]

To assess wider neighbourhood trends in activity (Objective 4), additional systematic observations were conducted at two nearby public spaces within the intervention neighbourhood that remained unchanged (Fig. 3). These sites were small amenity spaces located adjacent to housing and were commonly used informally by residents for relaxation, social interaction, and children’s play. In contrast to the intervention park, they lacked formal park infrastructure and were substantially smaller in size, and neither site was scheduled to receive improvements as part of the intervention.

These sites were included to assess whether increases in activity observed in the intervention park reflected displacement from nearby spaces. If the intervention primarily displaced existing activity, reductions in use would be expected at these nearby comparison sites following the intervention.

### Outcomes

#### Systematic behaviour observations

Behavioural outcomes were assessed using MOHAWk (Method for Observing pHysical Activity and Wellbeing), a validated systematic observation tool that captures three levels of PA (sedentary, walking, vigorous) and two additional wellbeing behaviours: Connect (social interactions) and Take Notice (taking notice of the environment). MOHAWk also records observer-estimated demographic characteristics, including age group (infant, child, teen, adult, older adult), gender (female or male), and ethnic group (white or non-white). Age groups are assigned based on observable characteristics such as general appearance, body size, clothing (e.g., school uniforms), and behaviour, and correspond approximately to the following life stages: infant (0–2 years), child (3–12 years), teen (13–19 years), adult (20–64 years), and older adult (65 + years). The tool has demonstrated high inter-rater reliability and criterion validity [37] and has been widely used in natural experimental studies of environmental interventions [32, 38–44].

Building on observations conducted at baseline (August 2018) and 15 months post-intervention (August 2021), observations were replicated at five years (63 months) post-intervention (August 2025), corresponding to seven years post-baseline.

At each time point, observations were conducted across six sites:

i. Two intervention sites (representing two areas within the park);
ii. Two matched comparison sites;
iii. Two nearby comparison sites.

For the four main sites (two intervention sites and two matched comparison sites), data were collected over two days (one weekday and one weekend day), with four one-hour observation periods per day, yielding eight observation hours per site (32 h per time point). Observations at the two nearby comparison sites followed the same two-day schedule, yielding 16 observation hours per time point.

To minimise bias related to time of day and day of week, observation schedules were counterbalanced across sites and rotated so that each site was observed equally across all time periods. The same observation structure was implemented at each time point, with observations conducted during comparable weeks of the year to account for seasonality (Additional file 4). This observation schedule has been shown to provide reliable estimates of PA in public spaces [37, 45].

Observations were conducted within predefined target areas representing comparable types of public space across sites (Additional file 5). Target areas were delineated using clearly identifiable physical boundaries (e.g., paths or edges of paved areas). All individuals observed within the target areas during each observation period were recorded. Observations took place regardless of weather conditions; observation periods with ≥ 30 min of precipitation were coded as “high precipitation” in line with MOHAWk guidance.

A total of five unique observers contributed to data collection across the study period, with three observers involved at each time point. Before data collection, the lead author (JSB) trained all observers using the MOHAWk manual and supervised practice observations. All observers had previously completed MOHAWk training in earlier studies and had demonstrated good-to-excellent inter-rater reliability when using the tool (intraclass correlation coefficients > 0.75). Formal inter-rater reliability was therefore not re-assessed for the present study, as all observers had already shown acceptable reliability and received additional supervised training before data collection.

#### Intercept surveys (self-reported outdoor space use)

Face-to-face intercept surveys were conducted to assess physical activity and wellbeing using a 10-item questionnaire (Additional file 6). For the purposes of this study, analyses focused on a single item assessing self-reported outdoor space use: “Other than passing by, I spent time in outdoor spaces within this neighbourhood boundary”, rated on a seven-point scale from ‘never’ to ‘everyday’. This item was used to capture broader neighbourhood outdoor space use rather than visits to the park specifically.

Surveys were conducted at baseline (June 2019) and five years post-intervention (August-September 2025). Surveys were attempted with all English-speaking adults (aged ≥ 18 years) within the intervention and comparison areas. To enhance representativeness, data collection took place both within and adjacent to green spaces as well as at nearby community locations (e.g., shops and GP surgeries) (Fig. 3). A quota-based sampling approach was used to ensure diversity across age, gender, and ethnicity.

### Logic model

Hypothesised causal pathways linking the intervention to behavioural outcomes were specified a priori using a logic model, which can be found in Additional file 7. The logic model was informed by the intervention co-design process, which helped identify the park features and behavioural outcomes most relevant to how residents were expected to use the redesigned park.

### Analyses

Analyses followed the approach used in our previous evaluation of the same intervention [32]. Any deviations from the published protocol are reported below.

#### Systematic behaviour observations

A difference-in-differences approach was used to estimate intervention effects. The unit of analysis was the one-hour observation period (counts per period per site). Analyses were conducted in Stata version 14.1. All statistical tests were two-sided, with statistical significance set at a priori alpha level of 0.05.

##### Primary outcome: walking

Intervention effects for the primary outcome were estimated using the interaction between group (intervention vs. comparison) and time point (baseline vs. follow-up). Multilevel mixed-effects negative binomial regression models were used to account for overdispersion and clustering at the site level. Models adjusted for day of week, time of day, and precipitation (binary).

In the protocol, time of day was specified as a categorical covariate. However, because some time categories were not observed on certain days (Additional file 4), this approach was not feasible. Time of day was therefore dichotomised as morning versus afternoon.

Although the protocol specified a combined outcome of walking and vigorous PA, these behaviours were analysed separately because they represent distinct forms of park-based PA that may respond differently to components of the intervention. This distinction is consistent with the study logic model (Additional file 7), which differentiates between walking (e.g., movement through the park) and vigorous PA (e.g., play and exercise) as behaviours influenced by different features of the park environment, and also maintains consistency with previous natural experimental studies using MOHAWk [38, 39]. Sensitivity analyses were conducted using a combined outcome of walking and vigorous PA.

Results are reported as incidence rate ratios (IRRs) with 95% confidence intervals (CIs). An IRR > 1 indicates higher counts in the intervention group relative to the comparison group, while an IRR < 1 indicates lower counts.

##### Secondary outcomes

The same analytical approach was applied to secondary outcomes, including vigorous PA, sedentary behaviour, Connect behaviours, and Take Notice behaviours.

##### Comparison of 15-month and five-year follow-ups

To assess how intervention effects evolved over time, outcomes at five years post-intervention were compared with those at the 15-month follow-up. Multilevel mixed-effects models for the primary and secondary outcomes were estimated with the 15-month follow-up specified as the reference category and the five-year follow-up as the comparison. This approach enabled assessment of whether intervention effects were sustained, attenuated, or amplified between the two post-intervention time points.

##### Wider neighbourhood trends

To assess wider neighbourhood trends, analyses were replicated in two ways. First, counts at the intervention sites were compared with those at nearby comparison sites within the same neighbourhood to assess whether observed changes could be explained by local population changes or displacement. Second, counts at the nearby comparison sites were compared with those at the matched comparison sites to assess whether activity patterns in the local neighbourhood diverged from wider trends.

One observation period at an nearby comparison site was excluded because it coincided with a nearby road closure during the morning school run, producing an unusually high count for one hour (250 people) that was not representative of typical use (range: 9–54 people per hour). Sensitivity analyses including this outlier were also conducted.

##### Exploratory outcomes: demographic characteristics

Exploratory analyses examined changes in the demographic characteristics of park users, including age group, gender, and ethnic group, using the same analytical approach described above. For age group analyses, infants, children, and teenagers were combined into a single ‘young people’ category.

#### Intercept surveys (self-reported outdoor space use)

A 2 × 2 between-subjects ANOVA was used to compare self-reported outdoor space use between intervention and comparison areas at follow-up relative to baseline.

## Results

### Systematic behaviour observations

At baseline, the intervention and comparison groups were broadly similar in observed age group, gender, and ethnic group proportions (Table 1). Although a slightly higher total number of people were observed at the comparison sites, the proportions of individuals engaging in each physical activity and wellbeing behaviour were comparable between groups.

**Table 1:** Baseline information on observation periods and sample in August 2018

	Comparison group (2 sites)	Intervention group (2 sites)
Observation periods - *n* (% of total)		
Total number	15 (1 missing)^a^	16
8-9am	3 (20%)	4 (25%)
10:30 − 11:30am	4 (26.7%)	4 (25%)
12:30 − 1:30pm	4 (26.7%)	4 (25%)
5-6pm	4 (26.7%)	4 (25%)
Wednesday	7 (46.7%)	8 (50%)
Thursday	4 (26.7%)	4 (25%)
Saturday	4 (26.7%)	4 (25%)
High precipitation	3 (20%)	2 (12.5%)
Sample size - n (median, IQR)		
Total number of people	538 (34, 11)	493 (30, 31)
Sample characteristics - n (% of total number of people)		
Non-white	129 (25%)	130 (27.5%)
White	387 (75%)	343 (72.5%)
Female	231 (42.9%)	188 (38.9%)
Male	308 (57.1%)	295 (61.1%)
Young person^b^	172 (32.0%)	126 (25.6%)
Adult	332 (61.7%)	346 (70.2%)
Older adult	34 (6.3%)	21 (4.3%)
MOHAWk behaviours – n (% of total number of people)		
Walking	454 (84.4%)	384 (77.9%)
Vigorous	75 (13.9%)	84 (17.0%)
Sedentary	104 (19.3%)	58 (11.8%)
Connect	197 (36.6%)	136 (27.6%)
Take Notice	20 (3.7%)	31 (6.3%)

^a^One observation period was missed at random at a comparison site due to researcher error

^b^Includes infants, children, and teenagers

Results from the mixed-effects negative binomial regression models comparing changes over time between intervention and comparison sites are presented in Table 2.

**Table 2:** Mixed-effects negative binomial regression results for primary and secondary outcomes

MOHAWk behaviour	Time point	Total counts (median per observation period)		Analysis period^c^	IRR	95% CI	*p*-value	Robust SE	Cluster random effect variance (site)
		Intervention group	Comparison group						
Walking^a^	Baseline	384 (18)	454 (27)	-	-	-	-	-	-
	15 months	698 (42)	262 (19)	-	-	-	-	-	-
	5 years	535 (27)	406 (26)	Baseline → 5 years	1.70	1.02–2.82	0.04*	0.44	0.02
				15 months → 5 years	0.48	0.37–0.64	< 0.001*	0.07	4.51e-30
Vigorous^b^	Baseline	84 (5)	75 (2)	-	-	-	-	-	-
	15 months	225 (9)	48 (2)	-	-	-	-	-	-
	5 years	143 (7.5)	78 (5)	Baseline → 5 years	1.46	0.74–2.87	0.27	0.50	0.12
				15 months → 5 years	0.40	0.22–0.74	0.004*	0.13	0.004
Sedentary^b^	Baseline	58 (3)	104 (6)	-	-	-	-	-	-
	15 months	241 (10)	50 (1)	-	-	-	-	-	-
	5 years	95 (3.5)	63 (2.5)	Baseline → 5 years	2.39	1.44–3.96	0.001*	0.62	0.07
				15 months → 5 years	0.16	0.01-2.00	0.16	0.21	0.28
Connect^b^	Baseline	136 (6)	197 (11)	-	-	-	-	-	-
	15 months	361 (21)	102 (6)	-	-	-	-	-	-
	5 years	269 (12.5)	188 (10.5)	Baseline → 5 years	2.08	1.34–3.23	0.001*	0.47	0.04
				15 months → 5 years	0.37	0.15–0.87	0.02*	0.16	0.001
Take Notice^b^	Baseline	31 (2)	20 (1)	-	-	-	-	-	-
	15 months	509 (23)	112 (7)	-	-	-	-	-	-
	5 years	140 (5.5)	17 (0)	Baseline → 5 years	4.77	2.54–8.97	< 0.001*	1.54	0.25
				15 months → 5 years	1.72	0.22–13.46	0.61	1.81	4.10e-33

Models adjusted for day of week, time of day, and precipitation

*CI* Confidence interval, *IRR* Incidence rate ratio, *SE* Standard error

^a^Primary outcome

^b^Secondary outcome

^c^Analysis period refers to the time interval used in the analysis, representing either change from baseline to 5-year follow-up or from 15-month follow-up to 5-year follow-up.

*Statistically significant at *p* < 0.05 (z-test, two-tailed)

#### Primary outcome: walking

**Fig. 4:**
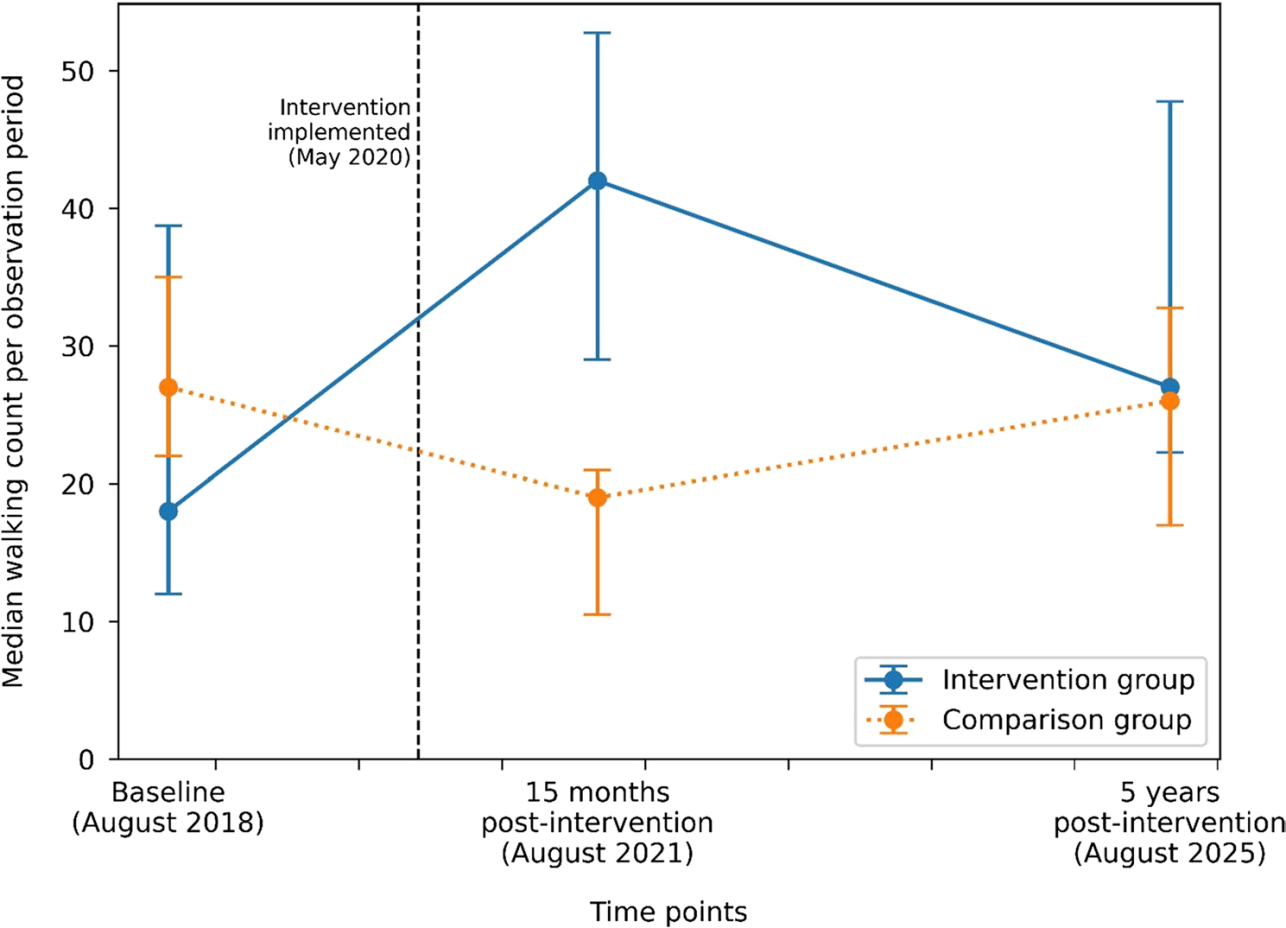
Median observed counts of walking (primary outcome) over time in intervention and comparison groups. *Points indicate medians per observation period; error bars represent interquartile ranges (IQRs)*

Compared with the comparison sites, the total number of people observed walking at the intervention sites increased significantly from baseline to five years post-intervention (IRR = 1.70, 95% CI 1.02–2.82, *p* = 0.04) (Table 2). Although median counts of walking were similar between groups at five years post-intervention, the significant intervention effect reflects a lower baseline counts in the intervention sites and a greater increase over time relative to the comparison sites (Fig. 4).

A sensitivity analysis combining walking and vigorous PA, as originally specified in the study protocol, did not change the direction or statistical significance of the effect (IRR = 1.70, 95% CI 1.10–2.62, *p* = 0.02).

#### Secondary outcomes

**Fig. 5:**
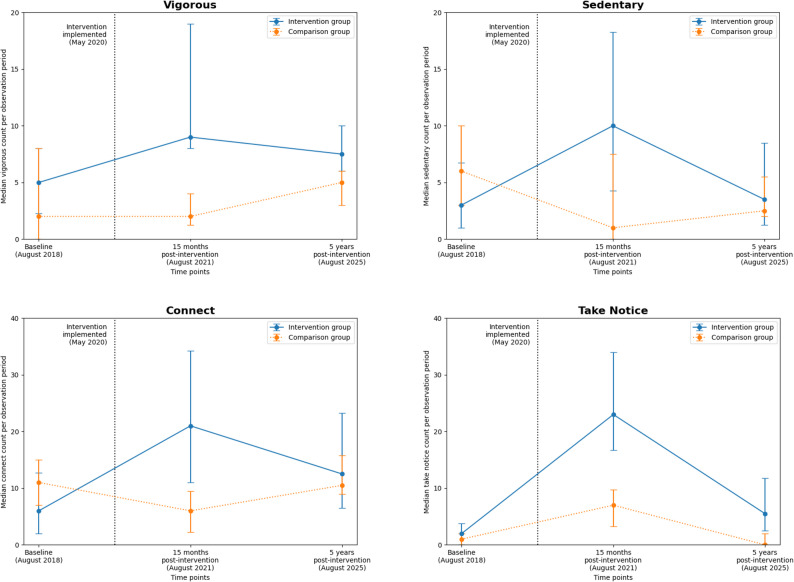
Median observed counts for secondary outcomes over time in intervention and comparison groups. *Points indicate medians per observation period; error bars represent interquartile ranges (IQRs)*

Vigorous PA increased at the intervention sites relative to the comparison sites between baseline and five years, although this difference was not statistically significant (IRR = 1.46, 95% CI 0.74–2.87, *p* = 0.27) (Fig. 5). In contrast, sedentary behaviour increased significantly more at the intervention sites than at the comparison sites (IRR = 2.39, 95% CI 1.44–3.96, *p* = 0.001) (Fig. 5). Connect behaviours also increased significantly at the intervention sites relative to the comparison sites (IRR = 2.08, 95% CI 1.34–3.23, *p* = 0.001) (Fig. 5). The largest relative increase was observed for Take Notice behaviours, which increased nearly fivefold at the intervention sites compared with the comparison sites (IRR = 4.77, 95% CI 2.54–8.97, *p* < 0.001) (Fig. 5).

#### Comparison of 15-month and five-year follow-ups

For walking, vigorous PA, sedentary, and Connect behaviours, IRRs comparing the 15-month and five-year follow-ups were below 1 (Table 2). This indicates that intervention effects generally attenuated over time, albeit still typically higher than baseline.

Although the IRR for Take Notice exceeded 1, suggesting a potential increase in effects between 15 months and five years post-intervention, this estimate was not statistically significant and was characterised by wide confidence intervals. Further, the increase was driven by a substantial decline in Take Notice counts at comparison sites rather than an increase at intervention sites (Table 2).

#### Wider neighbourhood trends

To determine whether observed effects reflected broader neighbourhood changes or displacement, activity at the intervention sites was compared with that at nearby comparison sites. From baseline to five years post-intervention, the total number of people observed at the intervention sites increased significantly relative to nearby comparison sites (IRR = 1.65, 95% CI 1.24–2.20, *p* = 0.001; Additional file 8), suggesting that the effects were specific to the intervention rather than driven by wider neighbourhood trends. A sensitivity analysis including one unusually busy observation period at an nearby comparison site (caused by a local road closure) yielded results in the same direction but of smaller magnitude and not statistically significant (IRR = 1.04, 95% CI 0.45–2.44, *p* = 0.92) (Additional file 8).

When nearby comparison sites were compared with the matched comparison sites, no significant differences in activity were observed (IRR = 0.99, 95% CI 0.73–1.34, *p* = 0.94; Additional file 8), indicating that activity patterns within the local neighbourhood broadly followed wider trends. A sensitivity analysis including the outlier observation period produced a larger effect size in the same direction, but this was not statistically significant (IRR = 0.67, 95% CI 0.29–1.57, *p* = 0.82) (Additional file 8).

#### Exploratory outcomes: demographic characteristics

Results of the exploratory analyses are reported in Additional file 9. From baseline to five years post-intervention, intervention sites showed substantial and statistically significant increases in the observed numbers of young people (infants, children, and teenagers) and non-white individuals relative to comparison sites. More modest but statistically significant increases were also observed for adults and males. No statistically significant changes were observed for older adults, females, or white individuals.

### Intercept surveys (self-reported outdoor space use)

A total of 449 participants completed the intercept surveys at baseline (2019; *n* = 217) and five years post-intervention (2025; *n* = 232) (Table 3). Baseline samples were broadly similar across intervention and comparison areas in terms of estimated age group, gender, and ethnic group (Additional file 10). Table 3 also presents the 12-month post-intervention survey data (reported elsewhere [32]) to enable comparison with baseline and 5-year follow-up values; these data are included for descriptive comparison only and were not analysed in the present study.

**Table 3:** Self-reported time spent in outdoor spaces in intervention and comparison groups

Time point	Intervention group		Comparison group	
	Mean (SD)	*n*	Mean (SD)	*n*
Baseline (2019)	1.37 (1.69)	100	2.87 (2.04)	117
12 months post-intervention (2021)^a^	4.52 (1.51)	225	4.59 (1.66)	218
5 years post-intervention (2025)	4.43 (1.68)	114	3.97 (1.98)	118

Self-reported time spent in outdoor spaces was measured using the question:“Other than passing by, I spent time in outdoor spaces within this neighbourhood boundary.”Response options were coded as: 0 = Never; 1 = Once in the last 4 weeks; 2 = 2–3 times in the last 4 weeks; 3 = Once a week; 4 = 2–3 times a week; 5 = Most days; 6 = Every day.

*SD* Standard deviation

^a^Survey data from the 12-month follow-up were not analysed in the present study.

There were significant differences in self-reported outdoor space use between groups (F(1,445) = 6.35, *p* = 0.01) and across time points (F(1,445) = 159.34, *p* < 0.001). Importantly, the interaction between group and time was significant (F(1,445) = 26.70, *p* < 0.001), indicating that increases in self-reported outdoor space use from baseline to five years post-intervention were significantly greater in the intervention area than in the comparison area.

Mean scores are reported on the original response scale, ranging from 0 (“Never”) to 6 (“Every day”). In the intervention area, the baseline mean of 1.37 corresponds approximately to visiting outdoor spaces once in the last four weeks, while the five-year follow-up mean of 4.43 corresponds to visiting around 2–3 times per week. In the comparison area, the baseline mean of 2.87 corresponds approximately to visiting around once per week, increasing to 3.97 at follow-up, equivalent to around 2–3 times per week.

## Discussion

### Key findings

This study shows that a co-designed, sustainable urban park intervention was associated with a significant increase in walking that was sustained five years post-intervention. While no significant increase in vigorous PA was observed, there were substantial and statistically significant increases in sedentary behaviour (reflecting time spent in the park), social interactions, and taking notice of the environment. Comparisons between the 15-month and five-year follow-ups indicated some attenuation of intervention effects over time. Comparisons with nearby sites indicated that the intervention effects were unlikely to be driven by displacement or wider neighbourhood-level changes in outdoor activity. The largest increases in park use were among non-white individuals and young people. Findings from the intercept surveys were consistent with the observational data, showing significantly greater increases in self-reported outdoor space use in the intervention area compared with the comparison area at five years post-intervention.

### Comparison with existing literature

To our knowledge, this is the first natural experimental study to evaluate the impacts of an urban park intervention after five years. Most previous evaluations have typically had follow-up periods of two years or less post-intervention [23], leaving uncertainty about whether park interventions can produce sustained changes in PA. In the present study, effect sizes were attenuated relative to those observed at 15 months, which may partly reflect diminishing novelty effects and the influence of the COVID-19 pandemic, during which green space use temporarily increased due to restrictions on indoor activities. Despite this attenuation, the persistence of significant effects on walking and other wellbeing behaviours at five years is notable, particularly given evidence that behavioural PA interventions often struggle to maintain effects beyond 15 months [46].

Our findings are consistent with natural experimental studies in low-income neighbourhoods in New York City [47], Melbourne [48], and Ghent [27], which have shown that high-quality park redevelopments increase park use and PA. By providing accessible, low-agency opportunities for recreation, social interaction, and psychological restoration, park interventions may help address longstanding inequities in access to safe, high-quality green space, which remains unevenly distributed across European cities [49]. Importantly, evidence suggests that residents of deprived areas and other marginalised groups may experience greater health benefits from equivalent exposure to green space [50], strengthening the case for park interventions as a strategy to reduce health inequalities.

The intervention was underpinned by an extensive co-design process. Our findings align with evidence that co-designed urban green spaces are more likely to increase use: a systematic review reported increased use in 109 of 120 co-designed green spaces, compared with declined use in nearly half of non-co-designed green spaces [51]. Embedding co-design may therefore have enhanced both initial uptake and longer-term engagement. This supports wider public health guidance indicating that interventions often fail when they lack contextual fit or acceptability due to insufficient community engagement [52].

Exploratory subgroup analyses showed particularly large increases in park use among non-white individuals. Previous research has identified safety concerns as a major barrier to park use among ethnic minority groups [53]. During the co-design process, local residents reported that the park felt unsafe, partly due to restricted visibility created by landscaped mounds. The redesign improved sightlines across the park, which may have enhanced perceived safety. Although perceived safety was not formally measured in the present study, these changes could plausibly have contributed to the observed increases in park use among non-white individuals.

Young people also demonstrated substantial increases in park use relative to comparison sites. This is consistent with previous evidence that park renovations often benefit children and young people more than older adults [25, 54–56]. One possible explanation for the absence of clear intervention effects among older adults is that this group is underrepresented among park users more broadly [57, 58], and they often prefer larger well-maintained parks [59]. Although older adults were actively involved in the co-design process, involvement in co-design may not necessarily translate into increased use if wider contextual factors (e.g., perceptions of safety) influence the extent to which different groups benefit from park improvements [23]. More targeted programming and design strategies may therefore be required to improve age inclusivity in smaller community parks.

### Strengths and limitations

Evaluating natural experiments is inherently challenging because researchers do not control the intervention [60, 61]. This study provides an example of how several methodological limitations commonly observed in natural experimental research can be addressed [22, 62]. Key strengths include the use of multiple matched comparison sites based on neighbourhood- and street-level characteristics; adjustment for key confounders (day of week, time of day, precipitation); a pre-registered protocol with a priori analysis plans; triangulation of objective behavioural observations with self-reported survey data; assessment of potential displacement using nearby comparison sites; and transparent reporting in line with standardised reporting checklists (Additional files 1 and 2). The study design also enabled replication across multiple follow-up time points, and similar methodological approaches have been applied in other natural experimental studies [38, 39, 41].

However, there are some limitations to acknowledge. Changes in the local population (e.g., residential turnover), wider neighbourhood development, or other contextual factors may have influenced park use independently of the intervention. Behavioural observation methods cannot distinguish whether increases in use reflect new users or more frequent use by existing residents, nor do they capture net population-level changes in PA or wellbeing [63]. In addition, the lack of a detailed process evaluation limits understanding of how and why the intervention produced its effects, and why observed effects appeared to attenuate over time. This limits the study’s contribution to development of new theoretical knowledge, which remains underdeveloped in this field [20]. Nonetheless, the study provides a rigorous assessment of how changes to the built environment influence behaviour within those spaces, which is useful knowledge for environmental intervention research [64].

### Implications for policy and practice

These findings have direct relevance for urban planners, local authorities, and policymakers. Co-designed, sustainable park interventions can generate long-term increases in PA, particularly among groups that are often underserved, while also delivering co-benefits such as improved flood management and biodiversity (reported elsewhere [65]). This evidence is especially timely given growing policy interest in urban interventions that simultaneously support population health and climate resilience [66]. By addressing repeated calls for long-term evaluations of urban green space interventions, this study provides policy-relevant evidence to inform efforts to create healthier and more equitable urban environments – an area in which decision-making is often undertaken in the absence of robust intervention-based evidence [67].

Importantly, because the intervention was implemented in a highly deprived neighbourhood, the sustained increases in park use and wellbeing-related behaviours highlight the potential of investing in high-quality green spaces as a strategy to reduce health inequalities. Environmental interventions such as park improvements typically require relatively low individual agency compared with many behaviour change interventions [68], making them more accessible across socioeconomic groups. In contrast, downstream PA interventions (e.g., activity trackers, apps, counselling) rely more heavily on personal resources such as time, income, and education, and are therefore often less effective among lower socioeconomic populations [69, 70]. Given that the park primarily serves nearby residents, it is plausible that these benefits accrued largely to the socioeconomically deprived local population. However, this cannot be confirmed, as individual socioeconomic status was not measured. This interpretation should also be treated cautiously in light of evidence on green gentrification, whereby park improvements may attract higher-income residents and displace lower-income populations, potentially limiting their impact on health inequalities [71].

### Future research

Although a logic model was developed to inform study design and outcome selection, future research should incorporate more detailed process evaluation to test and refine this model, in line with UK Medical Research Council (MRC) guidance on complex interventions [52]. Ongoing qualitative research with local residents is extending this work by developing a programme theory using realist evaluation [72], with the aim of explaining how and for whom intervention effects emerged, and how contextual factors shaped outcomes.

Further research is also needed to identify effective strategies for increasing park use among underrepresented groups, to ensure that park interventions do not inadvertently exclude certain populations. This is particularly important for girls and women, who were underrepresented in this study and consistently underrepresented in previous park-based research [23]. As intercept surveys captured data only from adults, while observational data indicated the largest increases in use among young people, there is a strong rationale for examining longer-term impacts of park interventions on children’s and adolescents’ PA. This is especially important given that a substantial proportion of moderate-to-vigorous PA during childhood occurs in green spaces [73]. Future studies could leverage existing longitudinal datasets with repeated measures (e.g., the #BeeWell survey [74]) to examine these longer-term effects.

Finally, this study highlights the importance of long-term evaluations in capturing the full value of urban green space interventions. Such research often extends beyond typical academic funding cycles [61], highlighting the need for more innovative and sustained funding mechanisms to support long-term natural experimental research.

## Conclusions

This natural experimental study provides rare and robust evidence that co-designed, sustainable urban park improvements can generate long-term increases in walking and other wellbeing-related behaviours in deprived urban areas, with effects persisting for at least five years. Urban green space interventions therefore represent an effective long-term strategy for increasing PA in areas with the greatest need. Further robust natural experimental studies are required to strengthen the evidence base for sustained investment in urban parks and to maximise long-term health and wellbeing benefits.

## Supplementary Information

Additional file 1. Completed STROBE checklist.

Additional file 2. Completed TIDieR checklist.

Additional file 3. Characteristics used to match intervention and comparison sites at baseline.

Additional file 4. Observation schedule.

Additional file 5. Target area boundaries.

Additional file 6. Intercept survey.

Additional file 7. Logic model.

Additional file 8. Wider neighbourhood trends analysis.

Additional file 9. Exploratory analyses.

Additional file 10. Intercept survey participant demographics.

## Data Availability

Data are only available on reasonable request from the corresponding author. All materials required to use MOHAWk are freely available in Benton et al. [34]. Any other study materials are available by directly contacting the corresponding author on reasonable request.

## Abbreviations

ANOVA: Analysis of variance
CI: Confidence interval
EU: European Union
GP: General practitioner
IQR: Interquartile range
IRR: Incidence rate ratio
LSOA: Lower Layer Super Output Area
MOHAWk: Method for Observing pHysical Activity and Wellbeing
MRC: Medical Research Council
PA: Physical activity
RCT: Randomised controlled trial
SD: Standard deviation
STROBE: Strengthening the Reporting of Observational Studies in Epidemiology
TIDieR: Template for Intervention Description and Replication
UK: United Kingdom
