# Supplementary material for: Long-term impacts of co-designed sustainable park improvements on physical activity and other wellbeing behaviours: a 7-year natural experimental study in a deprived urban area
Source: Int J Behav Nutr Phys Act. 2026 Apr 21;23:60. doi: 10.1186/s12966-026-01918-9 (PMC13237973; doi:10.1186/s12966-026-01918-9)
Supplement: Supplementary file 1 — Additional file 1. Completed STROBE checklist. [file 12966_2026_1918_MOESM1_ESM.docx]

**Additional file 1.** Completed STROBE checklist

|  | Item No. | Recommendation | Page  No. | Relevant text from manuscript |
| --- | --- | --- | --- | --- |
| Title and abstract | 1 | (*a*) Indicate the study’s design with a commonly used term in the title or the abstract | Title | ‘A 7-year natural experimental study’ |
|  |  | (*b*) Provide in the abstract an informative and balanced summary of what was done and what was found | Abstract | ‘Background: Creating or improving urban parks is a promising intervention for promoting physical activity (PA). However, robust natural experimental evidence of effectiveness remains limited, particularly regarding long-term impacts. We previously showed that co-designed, sustainable urban park improvements in a deprived UK area increased walking and other wellbeing-related behaviours up to 15 months post-intervention. We now examine whether these effects were sustained five years post-intervention (seven years after baseline).  Methods: Two intervention sites were matched to two comparison sites, with two additional nearby comparison sites included to assess wider neighbourhood trends. Outcomes were assessed using systematic observations at baseline (2018), 15 months post-intervention (2021), and five years post-intervention (2025). The primary outcome was change in the number of people walking; secondary outcomes included vigorous PA, sedentary behaviour, social interactions, and taking notice of the environment. Multilevel mixed-effects negative binomial regression models compared changes between intervention and comparison sites, adjusting for day of week, time of day, and precipitation. Additional analyses compared 15-month (2021) and five-year (2025) follow-ups to assess how intervention effects evolved over time. Intercept surveys assessed self-reported outdoor space use at baseline (2019; n = 217) and follow-up (2025; n = 232).  Results: From baseline to five years post-intervention, walking at the intervention sites significantly increased by 70% relative to the comparison sites (IRR = 1.70, 95% CI 1.02-2.82). Significant increases were also observed for sedentary behaviour, social interactions, and taking notice of the environment. Comparison of the 15-month and five-year follow-ups indicated some attenuation of intervention effects over time. Observations at nearby comparison sites suggested that these effects were not attributable to wider neighbourhood trends. The largest increases in observed park use were among young people and non-white ethnic groups. Intercept surveys corroborated the observational findings, showing significantly greater increases in self-reported outdoor space use in the intervention area.  Conclusions: Co-designed, sustainable urban park improvements can generate long-term increases in walking and other wellbeing-related behaviours in deprived urban areas, with effects persisting for at least five years. Urban green space interventions, which can target areas with the greatest need, therefore represent an effective long-term strategy for increasing PA.’ |
| Introduction | | | |  |
| Background/rationale | 2 | Explain the scientific background and rationale for the investigation being reported | 5-7 | Various points made in the Background  e.g. ‘…there are relatively few natural experimental studies evaluating the impacts of urban park interventions on PA’ |
| Objectives | 3 | State specific objectives, including any prespecified hypotheses | 8 | ‘The aim of this natural experimental study was to prospectively evaluate the long-term impacts of co-designed, sustainable park improvements in a deprived urban area in the UK. Specifically, the study assessed observed changes in walking and other wellbeing behaviours five years post-intervention (2025), i.e. seven years after baseline, relative to: (a) baseline (2018) and (b) 15-months post-intervention (2021).  The specific objectives were to compare observed changes at these timepoints in:   1. The total number of people walking (primary outcome); 2. The total number of people engaging in vigorous PA, sedentary behaviour, social interactions, and taking notice of the environment (secondary outcomes); 3. The demographic characteristics of park users (exploratory outcomes); 4. Activity at nearby comparison sites to assess wider neighbourhood trends;   A fifth objective was to assess changes in self-reported use of outdoor spaces using intercept surveys to triangulate with any observed changes in PA behaviour.’ |
| Methods | | | |  |
| Study design | 4 | Present key elements of study design early in the paper | 8 | ‘This was a controlled pre-post prospective natural experimental study’ |
| Setting | 5 | Describe the setting, locations, and relevant dates, including periods of recruitment, exposure, follow-up, and data collection | 9, 14 and 15 | ‘The intervention was a 1.4-hectare linear park opened in May 2020 in West Gorton, which is a highly deprived area of Manchester, UK - a large post-industrial city.’  ‘Building on observations conducted at baseline (August 2018) and 15 months post-intervention (August 2021), observations were replicated at five years (63 months) post-intervention (August 2025), corresponding to seven years post-baseline.  At each time point, observations were conducted across six sites:   1. Two intervention sites (representing two areas within the park); 2. Two matched comparison sites; 3. Two nearby comparison sites.   For the four main sites (two intervention sites and two matched comparison sites), data were collected over two days (one weekday and one weekend day), with four one-hour observation periods per day, yielding eight observation hours per site (32 hours per time point). Observations at the two nearby comparison sites followed the same two-day schedule, yielding 16 observation hours per time point.’  ‘Surveys were conducted at baseline (June 2019) and five years post-intervention (August-September 2025).’ |
| Participants | 6 | (*a*) *Cohort study*—Give the eligibility criteria, and the sources and methods of selection of participants. Describe methods of follow-up  *Case-control study*—Give the eligibility criteria, and the sources and methods of case ascertainment and control selection. Give the rationale for the choice of cases and controls  *Cross-sectional study*—Give the eligibility criteria, and the sources and methods of selection of participants | 15 and 16 | ‘Observations were conducted within predefined target areas representing comparable types of public space across sites (Additional file 5). Target areas were delineated using clearly identifiable physical boundaries (e.g., paths or edges of paved areas). All individuals observed within the target areas during each observation period were recorded. Observations took place regardless of weather conditions; observation periods with ≥30 minutes of precipitation were coded as “high precipitation” in line with MOHAWk guidance.’  ‘Surveys were attempted with all English-speaking adults (aged ≥18 years) within the intervention and comparison areas.’ |
|  |  | (*b*) *Cohort study*—For matched studies, give matching criteria and number of exposed and unexposed  *Case-control study*—For matched studies, give matching criteria and the number of controls per case | Page 12  Additional file 3 | ‘For the main analyses, two matched comparison sites in Greater Manchester were identified using a recently developed matching approach (36), based on fifteen key variables associated with PA (Additional file 3). First, neighbourhoods most similar to the intervention area were identified at the Lower Layer Super Output Area (LSOA) level using spatial data on population density, street connectivity, area-level deprivation, and greenness. Second, specific candidate sites within these neighbourhoods were identified through staged screening using Google Street View, followed by in-person site environmental audits and observations to assess footfall.’ |
| Variables | 7 | Clearly define all outcomes, exposures, predictors, potential confounders, and effect modifiers. Give diagnostic criteria, if applicable | Pages 12 and 15  Additional file 3 | ‘For the main analyses, two matched comparison sites in Greater Manchester were identified using a recently developed matching approach (36), based on fifteen key variables associated with PA (Additional file 3). First, neighbourhoods most similar to the intervention area were identified at the Lower Layer Super Output Area (LSOA) level using spatial data on population density, street connectivity, area-level deprivation, and greenness. Second, specific candidate sites within these neighbourhoods were identified through staged screening using Google Street View, followed by in-person site environmental audits and observations to assess footfall.’  ‘Observations took place regardless of weather conditions; observation periods with ≥30 minutes of precipitation were coded as “high precipitation” in line with MOHAWk guidance.’ |
| Data sources/ measurement | 8* | For each variable of interest, give sources of data and details of methods of assessment (measurement). Describe comparability of assessment methods if there is more than one group | 14 and 15 | ‘Behavioural outcomes were assessed using MOHAWk (Method for Observing pHysical Activity and Wellbeing), a validated systematic observation tool that captures three levels of PA (sedentary, walking, vigorous) and two additional wellbeing behaviours: Connect (social interactions) and Take Notice (taking notice of the environment). MOHAWk also records observer-estimated demographic characteristics, including age group (infant, child, teen, adult, older adult), gender (female or male), and ethnic group (white or non-white). Age groups are assigned based on observable characteristics such as general appearance, body size, clothing (e.g., school uniforms), and behaviour, and correspond approximately to the following life stages: infant (0-2 years), child (3-12 years), teen (13-19 years), adult (20-64 years), and older adult (65+ years).’  ‘A total of five unique observers contributed to data collection across the study period, with three observers involved at each time point. Before data collection, the lead author (JSB) trained all observers using the MOHAWk manual and supervised practice observations. All observers had previously completed MOHAWk training in earlier studies and had demonstrated good-to-excellent inter-rater reliability when using the tool (intraclass correlation coefficients > 0.75). Formal inter-rater reliability was therefore not re-assessed for the present study, as all observers had already shown acceptable reliability and received additional supervised training before data collection.’ |
| Bias | 9 | Describe any efforts to address potential sources of bias | 31 and 32 | ‘This study provides an example of how several methodological limitations commonly observed in natural experimental research can be addressed (22, 62). Key strengths include the use of multiple matched comparison sites based on neighbourhood- and street-level characteristics; adjustment for key confounders (day of week, time of day, precipitation); a pre-registered protocol with a priori analysis plans; triangulation of objective behavioural observations with self-reported survey data; assessment of potential displacement using nearby comparison sites; and transparent reporting in line with standardised reporting checklists (Additional files 1 and 2). The study design also enabled replication across multiple follow-up time points, and similar methodological approaches have been applied in other natural experimental studies (38, 39, 41).’ |
| Study size | 10 | Explain how the study size was arrived at | 15 | ‘The same observation structure was implemented at each time point, with observations conducted during comparable weeks of the year to account for seasonality (Additional file 4). This observation schedule has been shown to provide reliable estimates of PA in public spaces (37, 45).’ |

Continued on next page

| Quantitative variables | 11 | Explain how quantitative variables were handled in the analyses. If applicable, describe which groupings were chosen and why | 17 | ‘The unit of analysis was the one-hour observation period (counts per period per site).’ |
| --- | --- | --- | --- | --- |
| Statistical methods | 12 | (*a*) Describe all statistical methods, including those used to control for confounding | 17 and 18 | ‘Intervention effects for the primary outcome were estimated using the interaction between group (intervention vs. comparison) and time point (baseline vs. follow-up). Multilevel mixed-effects negative binomial regression models were used to account for overdispersion and clustering at the site level. Models adjusted for day of week, time of day, and precipitation (binary).  In the protocol, time of day was specified as a categorical covariate. However, because some time categories were not observed on certain days (Additional file 4), this approach was not feasible. Time of day was therefore dichotomised as morning versus afternoon.  Although the protocol specified a combined outcome of walking and vigorous PA, these behaviours were analysed separately because they represent distinct forms of park-based PA that may respond differently to components of the intervention. This distinction is consistent with the study logic model (Additional file 7), which differentiates between walking (e.g., movement through the park) and vigorous PA (e.g., play and exercise) as behaviours influenced by different features of the park environment, and also maintains consistency with previous natural experimental studies using MOHAWk (38, 39). Sensitivity analyses were conducted using a combined outcome of walking and vigorous PA.  Results are reported as incidence rate ratios (IRRs) with 95% confidence intervals (CIs). An IRR >1 indicates higher counts in the intervention group relative to the comparison group, while an IRR <1 indicates lower counts.’ |
|  |  | (*b*) Describe any methods used to examine subgroups and interactions | 19 | ‘Exploratory analyses examined changes in the demographic characteristics of park users, including age group, gender, and ethnic group, using the same analytical approach described above. For age group analyses, infants, children, and teenagers were combined into a single ‘young people’ category.’ |
|  |  | (*c*) Explain how missing data were addressed | N/A | N/A |
|  |  | (*d*) *Cohort study*—If applicable, explain how loss to follow-up was addressed  *Case-control study*—If applicable, explain how matching of cases and controls was addressed  *Cross-sectional study*—If applicable, describe analytical methods taking account of sampling strategy | N/A | N/A |
|  |  | (*e*) Describe any sensitivity analyses | 17-19 | ‘Although the protocol specified a combined outcome of walking and vigorous PA, these behaviours were analysed separately because they represent distinct forms of park-based PA that may respond differently to components of the intervention. This distinction is consistent with the study logic model (Additional file 7), which differentiates between walking (e.g., movement through the park) and vigorous PA (e.g., play and exercise) as behaviours influenced by different features of the park environment, and also maintains consistency with previous natural experimental studies using MOHAWk (38, 39). Sensitivity analyses were conducted using a combined outcome of walking and vigorous PA.’  ‘One observation period at an nearby comparison site was excluded because it coincided with a nearby road closure during the morning school run, producing an unusually high count for one hour (250 people) that was not representative of typical use (range: 9-54 people per hour). Sensitivity analyses including this outlier were also conducted.’ |
| Results | | | | |
| Participants | 13* | (a) Report numbers of individuals at each stage of study—eg numbers potentially eligible, examined for eligibility, confirmed eligible, included in the study, completing follow-up, and analysed | Tables 1-3 |  |
|  |  | (b) Give reasons for non-participation at each stage | N/A |  |
|  |  | (c) Consider use of a flow diagram | N/A |  |
| Descriptive data | 14* | (a) Give characteristics of study participants (eg demographic, clinical, social) and information on exposures and potential confounders | Table 2  Additional files 9 and 10 |  |
|  |  | (b) Indicate number of participants with missing data for each variable of interest | N/A |  |
|  |  | (c) *Cohort study*—Summarise follow-up time (eg, average and total amount) | 9 and 16 | ‘‘The intervention was a 1.4-hectare linear park opened in May 2020 in West Gorton, which is a highly deprived area of Manchester, UK - a large post-industrial city.’  ‘Building on observations conducted at baseline (August 2018) and 15 months post-intervention (August 2021), observations were replicated at five years (63 months) post-intervention (August 2025), corresponding to seven years post-baseline. At each time point, observations were conducted across six sites: two intervention sites, two matched comparison sites, and two unchanged nearby sites.’  ‘Surveys were conducted at baseline (June 2019) and five years post-intervention (August-September 2025).’ |
| Outcome data | 15* | *Cohort study*—Report numbers of outcome events or summary measures over time | Tables 2 and 3  Additional files 8 and 9 |  |
|  |  | *Case-control study—*Report numbers in each exposure category, or summary measures of exposure | N/A | N/A |
|  |  | *Cross-sectional study—*Report numbers of outcome events or summary measures | N/A | N/A |
| Main results | 16 | (*a*) Give unadjusted estimates and, if applicable, confounder-adjusted estimates and their precision (eg, 95% confidence interval). Make clear which confounders were adjusted for and why they were included | 19-29  Tables 2 and 3  Additional files 8 and 9 | Various results presented in the Results section, for example, the primary outcome results are reported as follows:  ‘Compared with the comparison sites, the total number of people observed walking at the intervention sites increased significantly from baseline to five years post-intervention (IRR = 1.70, 95% CI 1.02-2.82, p = 0.04) (Table 2). Although median counts of walking were similar between groups at five years post-intervention, the significant intervention effect reflects a lower baseline counts in the intervention sites and a greater increase over time relative to the comparison sites (Figure 4).  A sensitivity analysis combining walking and vigorous PA, as originally specified in the study protocol, did not change the direction or statistical significance of the effect (IRR = 1.70, 95% CI 1.10-2.62, p = 0.02).’ |
|  |  | (*b*) Report category boundaries when continuous variables were categorized | N/A | N/A |
|  |  | (*c*) If relevant, consider translating estimates of relative risk into absolute risk for a meaningful time period | N/A | N/A |

Continued on next page

| Other analyses | 17 | Report other analyses done—eg analyses of subgroups and interactions, and sensitivity analyses | 21-23  Additional file 8 | ‘A sensitivity analysis combining walking and vigorous PA, as originally specified in the study protocol, did not change the direction or statistical significance of the effect (IRR = 1.70, 95% CI 1.10-2.62, p = 0.02).’  ‘A sensitivity analysis including one unusually busy observation period at an nearby comparison site (caused by a local road closure) yielded results in the same direction but of smaller magnitude and not statistically significant (IRR = 1.04, 95% CI 0.45-2.44, p = 0.92) (Additional file 8).’  ‘A sensitivity analysis including the outlier observation period produced a larger effect size in the same direction, but this was not statistically significant (IRR = 0.67, 95% CI 0.29-1.57, p = 0.82) (Additional file 8).’ |
| --- | --- | --- | --- | --- |
| Discussion | | | | |
| Key results | 18 | Summarise key results with reference to study objectives | 29 | ‘This study shows that a co-designed, sustainable urban park intervention was associated with a significant increase in walking that was sustained five years post-intervention. While no significant increase in vigorous PA was observed, there were substantial and statistically significant increases in sedentary behaviour (reflecting time spent in the park), social interactions, and taking notice of the environment. Comparisons between the 15-month and five-year follow-ups indicated some attenuation of intervention effects over time. Comparisons with nearby sites indicated that the intervention effects were unlikely to be driven by displacement or wider neighbourhood-level changes in outdoor activity. The largest increases in park use were among non-white individuals and young people. Findings from the intercept surveys were consistent with the observational data, showing significantly greater increases in self-reported outdoor space use in the intervention area compared with the comparison area at five years post-intervention.’ |
| Limitations | 19 | Discuss limitations of the study, taking into account sources of potential bias or imprecision. Discuss both direction and magnitude of any potential bias | 32 | 'However, there are some limitations to acknowledge. Changes in the local population (e.g., residential turnover), wider neighbourhood development, or other contextual factors may have influenced park use independently of the intervention. Behavioural observation methods cannot distinguish whether increases in use reflect new users or more frequent use by existing residents, nor do they capture net population-level changes in PA or wellbeing (63). In addition, the lack of a detailed process evaluation limits understanding of how and why the intervention produced its effects, and why observed effects appeared to attenuate over time. This limits the study’s contribution to development of new theoretical knowledge, which remains underdeveloped in this field (20). Nonetheless, the study provides a rigorous assessment of how changes to the built environment influence behaviour within those spaces, which is useful knowledge for environmental intervention research (64). |
| Interpretation | 20 | Give a cautious overall interpretation of results considering objectives, limitations, multiplicity of analyses, results from similar studies, and other relevant evidence | 29-31 | ‘To our knowledge, this is the first natural experimental study to evaluate the impacts of an urban park intervention after five years. Most previous evaluations have typically had follow-up periods of two years or less post-intervention (23), leaving uncertainty about whether park interventions can produce sustained changes in PA. In the present study, effect sizes were attenuated relative to those observed at 15 months, which may partly reflect diminishing novelty effects and the influence of the COVID-19 pandemic, during which green space use temporarily increased due to restrictions on indoor activities. Despite this attenuation, the persistence of significant effects on walking and other wellbeing behaviours at five years is notable, particularly given evidence that behavioural PA interventions often struggle to maintain effects beyond 15 months (46).  Our findings are consistent with natural experimental studies in low-income neighbourhoods in New York City (47), Melbourne (48), and Ghent (27), which have shown that high-quality park redevelopments increase park use and PA. By providing accessible, low-agency opportunities for recreation, social interaction, and psychological restoration, park interventions may help address longstanding inequities in access to safe, high-quality green space, which remains unevenly distributed across European cities (49). Importantly, evidence suggests that residents of deprived areas and other marginalised groups may experience greater health benefits from equivalent exposure to green space (50), strengthening the case for park interventions as a strategy to reduce health inequalities.  The intervention was underpinned by an extensive co-design process. Our findings align with evidence that co-designed urban green spaces are more likely to increase use: a systematic review reported increased use in 109 of 120 co-designed green spaces, compared with declined use in nearly half of non-co-designed green spaces (51). Embedding co-design may therefore have enhanced both initial uptake and longer-term engagement. This supports wider public health guidance indicating that interventions often fail when they lack contextual fit or acceptability due to insufficient community engagement (52).  Exploratory subgroup analyses showed particularly large increases in park use among non-white individuals. Previous research has identified safety concerns as a major barrier to park use among ethnic minority groups (53). During the co-design process, local residents reported that the park felt unsafe, partly due to restricted visibility created by landscaped mounds. The redesign improved sightlines across the park, which may have enhanced perceived safety. Although perceived safety was not formally measured in the present study, these changes could plausibly have contributed to the observed increases in park use among non-white individuals.  Young people also demonstrated substantial increases in park use relative to comparison sites. This is consistent with previous evidence that park renovations often benefit children and young people more than older adults (25, 54-56). One possible explanation for the absence of clear intervention effects among older adults is that this group is underrepresented among park users more broadly (57, 58), and they often prefer larger well-maintained parks (59). Although older adults were actively involved in the co-design process, involvement in co-design may not necessarily translate into increased use if wider contextual factors (e.g., perceptions of safety) influence the extent to which different groups benefit from park improvements (23). More targeted programming and design strategies may therefore be required to improve age inclusivity in smaller community parks.’ |
| Generalisability | 21 | Discuss the generalisability (external validity) of the study results | 33 | ‘Although a logic model was developed to inform study design and outcome selection, future research should incorporate more detailed process evaluation to test and refine this model, in line with UK Medical Research Council (MRC) guidance on complex interventions (52). Ongoing qualitative research with local residents is extending this work by developing a programme theory using realist evaluation (72), with the aim of explaining how and for whom intervention effects emerged, and how contextual factors shaped outcomes.’ |
| Other information | |  | | |
| Funding | 22 | Give the source of funding and the role of the funders for the present study and, if applicable, for the original study on which the present article is based | 36 | ‘This research was supported by the European Union's Horizon 2020 research and innovation programme (grant number 730283). JSB is funded by a Leverhulme Trust Early Career Fellowship (ECF-2024-603). DPF is a National Institute for Health and Care Research (NIHR) Senior Investigator (NIHR305827). The views expressed in this article are those of the author(s) and not necessarily those of the funders.’ |

*Give information separately for cases and controls in case-control studies and, if applicable, for exposed and unexposed groups in cohort and cross-sectional studies.

**Note:** An Explanation and Elaboration article discusses each checklist item and gives methodological background and published examples of transparent reporting. The STROBE checklist is best used in conjunction with this article (freely available on the Web sites of PLoS Medicine at http://www.plosmedicine.org/, Annals of Internal Medicine at http://www.annals.org/, and Epidemiology at http://www.epidem.com/). Information on the STROBE Initiative is available at www.strobe-statement.org.
