# Supplementary material for: Long-term impacts of co-designed sustainable park improvements on physical activity and other wellbeing behaviours: a 7-year natural experimental study in a deprived urban area
Source: Int J Behav Nutr Phys Act. 2026 Apr 21;23:60. doi: 10.1186/s12966-026-01918-9 (PMC13237973; doi:10.1186/s12966-026-01918-9)
Supplement: Supplementary file 3 — Additional file 3. Characteristics used to match intervention and comparison sites at baseline. [file 12966_2026_1918_MOESM3_ESM.docx]

**Additional file 2.** Characteristics used to match intervention and comparison sites at baseline

| **Domain** | **Intervention sites** | **Comparison site 1 (Pendleton)** | **Comparison site 2 (Ardwick)** |
| --- | --- | --- | --- |
| **LSOA level** | | | |
| Index of Multiple Deprivation (IMD) rank | 78 | 70 | 76 |
| Population density (persons/ha) | 27 | 49 | 46 |
| Normalised Difference Vegetation Index (NDVI) | 0.3 | 0.3 | 0.3 |
| Street intersection density^1^ | 14 | 17 | 15 |
| **Postcode level** | | | |
| Walk Score | 54 | 58 | 65 |
| Regeneration context | Yes (refurbishment and new build) | Yes (refurbishment and new build) | Yes (refurbishment) |
| **Site level** | | | |
| Location | Relatively cut-off | Relatively cut-off | Adjacent to A-Road |
| Undulating landscape/ mounds | Yes | Yes | No |
| Noise (qualitative assessment) | Low | Moderate | Moderate |
| Adjacent land uses | Residential and light industry | Residential and light industry | Residential and commercial |
| Site width | Relatively wide (c.50 meters) | Narrow (15 meters) | Relatively wide (c.50 meters) |
| Biodiversity | High | Middle to high | Low |
| Children’s play provision | Yes | Yes | No |
| Number of benches | 3 | 4 | None |
| **Footfall** | | | |
| Mean persons per 15 minutes | 8 | 7 | 13 |
| ^1^ Number of 3-way junctions standardised by LSOA area | | | |
