# Supplementary material for: Long-term impacts of co-designed sustainable park improvements on physical activity and other wellbeing behaviours: a 7-year natural experimental study in a deprived urban area
Source: Int J Behav Nutr Phys Act. 2026 Apr 21;23:60. doi: 10.1186/s12966-026-01918-9 (PMC13237973; doi:10.1186/s12966-026-01918-9)
Supplement: Supplementary file 4 — Additional file 4. Observation schedule. [file 12966_2026_1918_MOESM4_ESM.docx]

**Additional file 3.** Observation schedule

|  | **Baseline observation schedule - August/September 2018** | | | | | | | | | | |
| --- | --- | --- | --- | --- | --- | --- | --- | --- | --- | --- | --- |
|  | **Monday** | **Tuesday** | **Wednesday** | | **Thursday** | | **Friday** | **Saturday**^1^ | | **Sunday** |  |
|  | **20^th^** | **21^st^** | **22^nd^** | | **23^rd^** | | **24^th^** | **25^th^** | | **26^th^** |  |
| 8am |  |  | Nearby comparison site 1 | Comparison site 2 |  | Comparison site 1 |  |  | |  |  |
| 10.30am |  |  | Nearby comparison site 1 | Comparison site 2 |  | Comparison site site 1 |  |  |  |  |  |
| 12.30pm |  |  | Intervention 1 | Comparison site 2 |  | Nearby comparison site 1 |  |  |  |  |  |
| 5.15pm |  |  | Intervention 1 | Comparison site 2 |  | Nearby comparison site 1 |  |  |  |  |  |
|  | **27^th^ (Bank Holiday)** | **28^th^** | **29^th^** | | **30^th^** | | **31^st^** | **1^st^** | | **2^nd^** |  |
| 8am |  |  |  | Intervention 2 |  | Intervention 1 |  |  | Comparison site 2 |  |  |
| 10.30am |  |  |  | Intervention 2 |  | Intervention 1 |  |  | Comparison site 2 |  |  |
| 12.30pm |  |  |  | Nearby comparison site 2 |  | Comparison site 1 |  |  | Intervention 2 |  |  |
| 5.15pm |  |  |  | Nearby comparison site 2 |  | Comparison site 1 |  |  | Intervention 2 |  |  |
|  | *End of Manchester schools summer holidays* | | | | | | | | | | |
|  | **3^rd^** | **4^th^** | **5^th^** | | **6^th^** | | **7^th^** | **8^th^** | | **9^th^** |  |
| 8am |  |  | Nearby comparison site 2 | Comparison site 1 |  | |  | Intervention 2 | Nearby comparison site 2 |  |  |
| 10.30am |  |  | Nearby comparison site 2 | Comparison site 1 |  |  |  | Intervention 2 | Nearby comparison site 2 |  |  |
| 12.30pm |  |  | Intervention 2 | Comparison site 1 |  |  |  | Nearby comparison site 2 | Comparison site 2 |  |  |
| 5.15pm |  |  | Intervention 2 | Comparison site 1 |  |  |  | Nearby comparison site 2 | Comparison site 2 |  |  |
|  | **10^th^** | **11^th^** | **12^th^** | | **13^th^** | | **14^th^** | **15^th^** | | **16^th^** |  |
| 8am |  |  | Intervention 1 |  | Nearby comparison site 1 |  |  |  | |  |  |
| 10.30am |  |  | Intervention 1 |  | Nearby comparison site 1 |  |  |  |  |  |  |
| 12.30pm |  |  | Nearby comparison site 1 |  | Intervention 1 |  |  |  |  |  |  |
| 5.15pm |  |  | Nearby comparison site 1 |  | Intervention 1 |  |  |  |  |  |  |
| ^1^ Saturday observation start: 9.30am, 11.30am, 1.30pm, 4.00pm  Each column within a given day represents a single observer | | | | | | | | | | | |
