# Supplementary material for: Long-term impacts of co-designed sustainable park improvements on physical activity and other wellbeing behaviours: a 7-year natural experimental study in a deprived urban area
Source: Int J Behav Nutr Phys Act. 2026 Apr 21;23:60. doi: 10.1186/s12966-026-01918-9 (PMC13237973; doi:10.1186/s12966-026-01918-9)
Supplement: Supplementary file 5 — Additional file 5. Target area boundaries. [file 12966_2026_1918_MOESM5_ESM.pdf]

Intervention sites 1 & 2

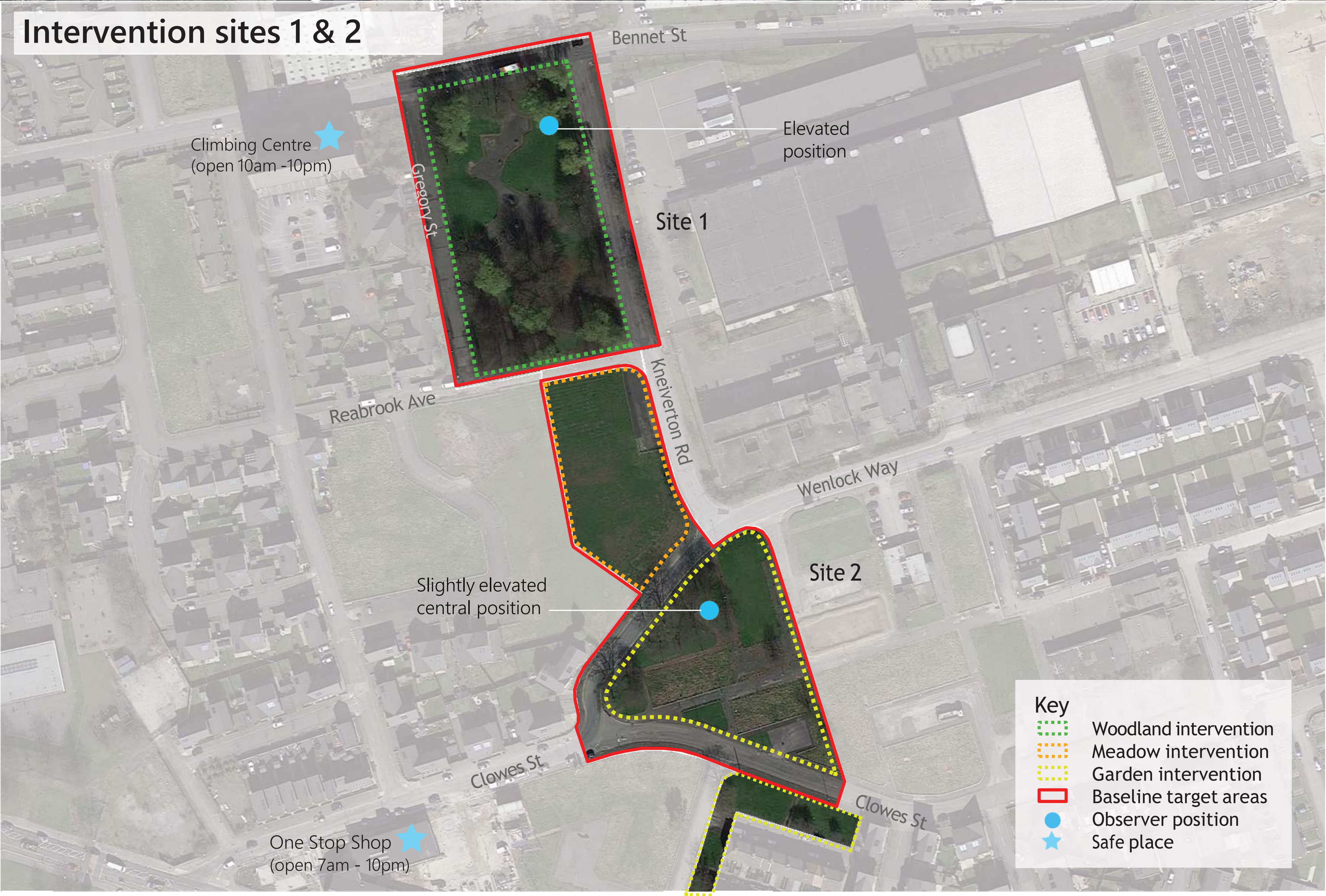

Comparison site 1

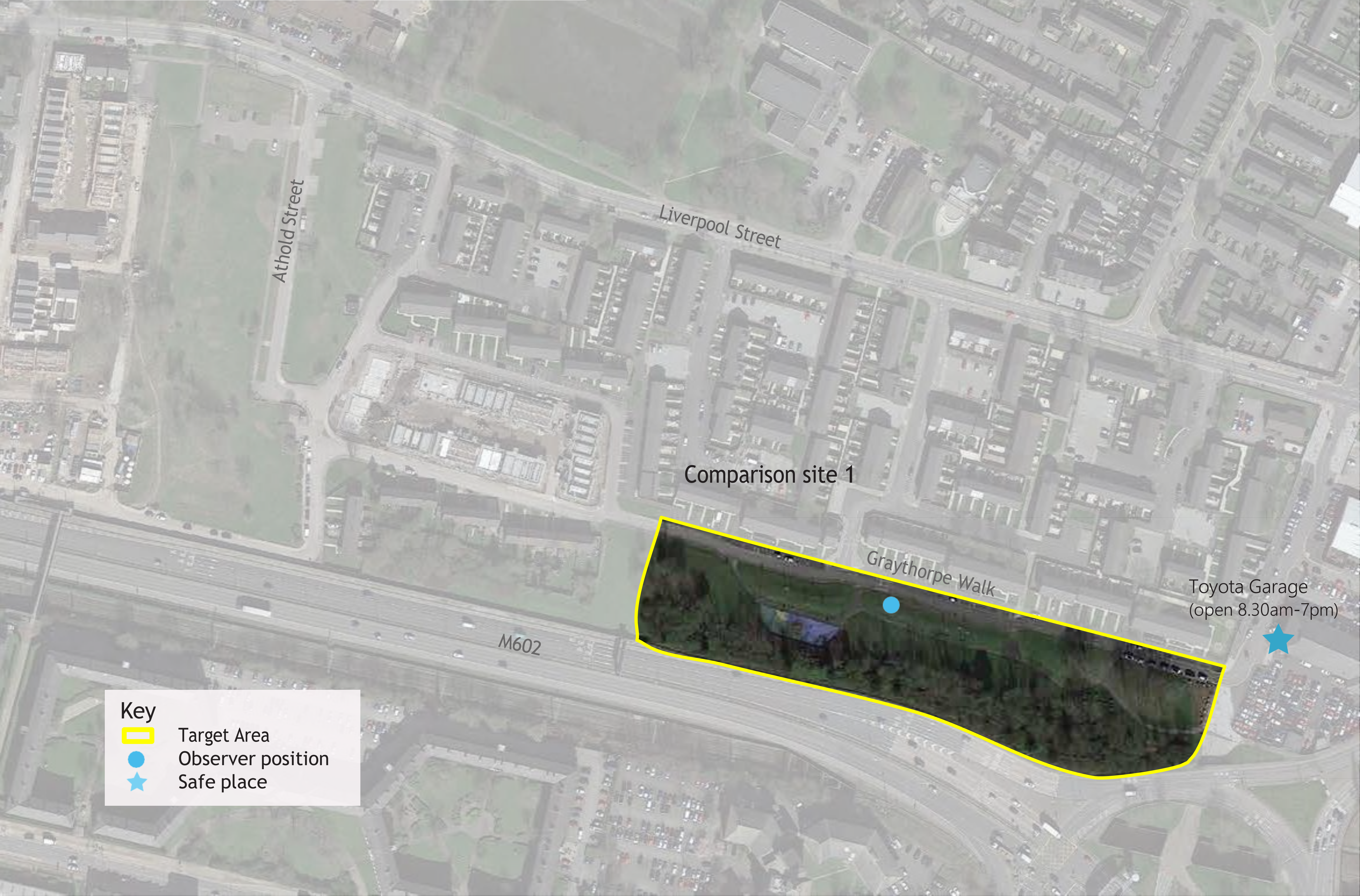

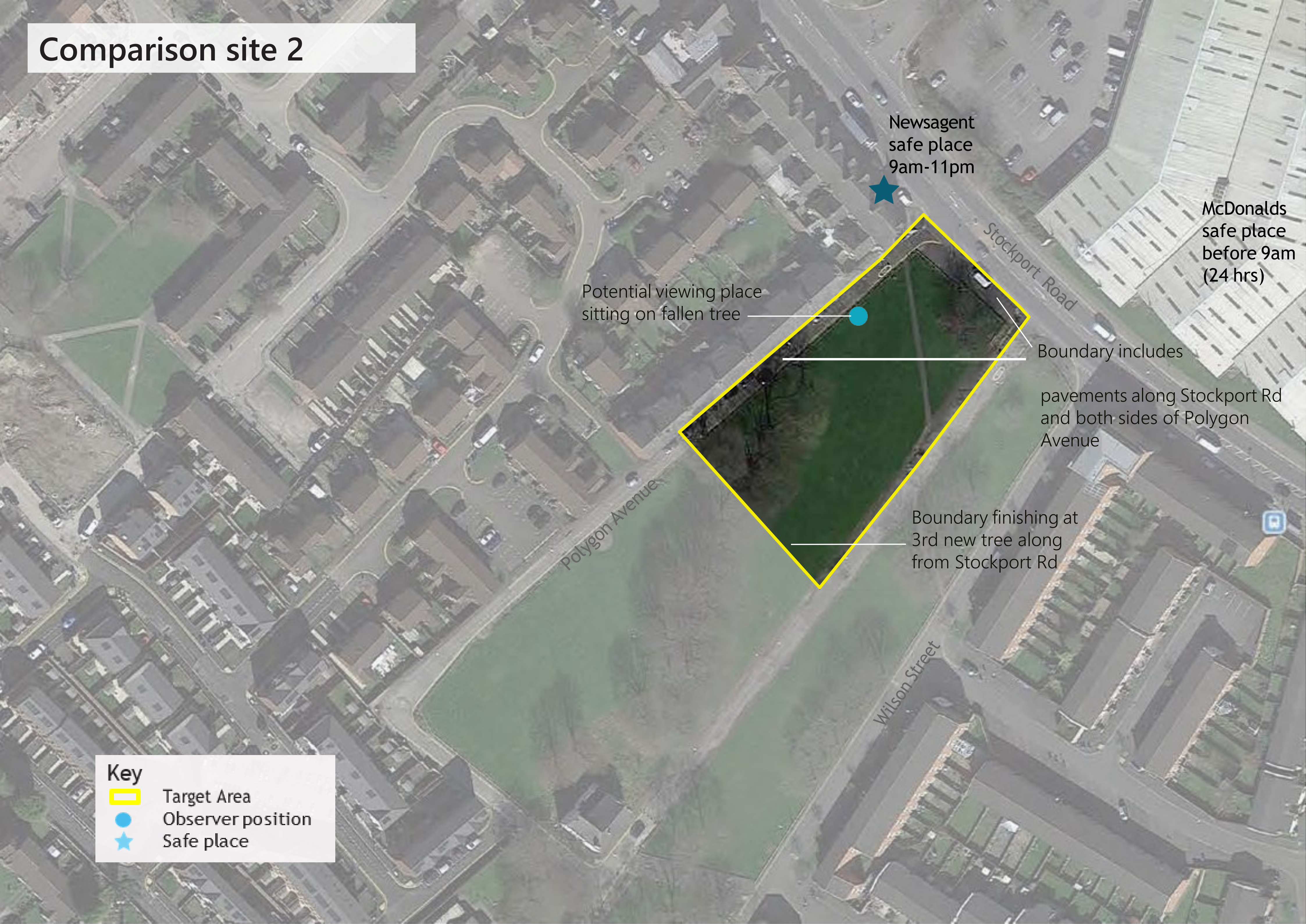
Comparison site 2
Newsagent safe place 9am-11pm
McDonalds safe place before 9am (24 hrs)
Stockport Road
Potential viewing place sitting on fallen tree
Boundary includes pavements along Stockport Rd and both sides of Polygon Avenue
Polygon Avenue
Boundary finishing at 3rd new tree along from Stockport Rd
Wilson Street
Key
Target Area
Observer position
Safe place
