# Supplementary material for: Long-term impacts of co-designed sustainable park improvements on physical activity and other wellbeing behaviours: a 7-year natural experimental study in a deprived urban area
Source: Int J Behav Nutr Phys Act. 2026 Apr 21;23:60. doi: 10.1186/s12966-026-01918-9 (PMC13237973; doi:10.1186/s12966-026-01918-9)
Supplement: Supplementary file 6 — Additional file 6. Intercept survey. [file 12966_2026_1918_MOESM6_ESM.docx]

**
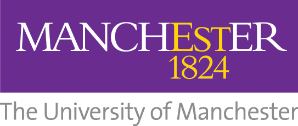
Neighbourhood wellbeing**

*Before we proceed could we check:*

- *That you are 18 or over?*
- *That you live within this printed boundary? (show printed map)*

Please indicate how much you agree or disagree with the following statements:

|  | **Strongly disagree** | **Disagree** | **Neither agree nor disagree** | **Agree** | **Strongly Agree** |  | **Not sure** |
| --- | --- | --- | --- | --- | --- | --- | --- |
| There are always opportunities to socialise in my neighbourhood | □ | □ | □ | □ | □ |  | □ |
| People in my neighbourhood treat me fairly | □ | □ | □ | □ | □ |  | □ |
| I feel safe walking alone in my neighbourhood at night | □ | □ | □ | □ | □ |  | □ |
| People in my neighbourhood are good at organising local events | □ | □ | □ | □ | □ |  | □ |
| I am suspicious of other people in my neighbourhood | □ | □ | □ | □ | □ |  | □ |
| I often hear of, or see neighbours pulling together to support each other | □ | □ | □ | □ | □ |  | □ |
| People in my neighbourhood can influence local issues | □ | □ | □ | □ | □ |  | □ |

**Not at all Completely**

**happy happy**

|  | **0** | **1** | **2** | **3** | **4** | **5** | **6** | **7** | **8** | **9** | **10** |  | **Not sure** |
| --- | --- | --- | --- | --- | --- | --- | --- | --- | --- | --- | --- | --- | --- |
| Overall, how happy did you feel yesterday? | □ | □ | □ | □ | □ | □ | □ | □ | □ | □ | □ |  | □ |

|  | **Never** | **Once in the last 4 weeks** | **2 to 3 times in the last 4 weeks** | **Once a week** | **2 to 3 times a week** | **Most days** | **Every day** |  | **Not sure** |
| --- | --- | --- | --- | --- | --- | --- | --- | --- | --- |
| Other than passing by, I spent time in outdoor spaces within the neighbourhood boundary | □ | □ | □ | □ | □ | □ | □ |  | □ |

| Approximately how many years of education have you completed **after age 16** (full-time or part-time)? |
| --- |
| ___________________________ years |

*Surveyors to complete*

**Participant number:** ______________________

| **Estimates** | | |
| --- | --- | --- |
| **Gender** | Male □ | Female □ |
| **Age group** | Adult □ | Older adult (~60+ years) □ |
| **Ethnic group** | White □ | Non-white □ |

| **Intercept location** | |
| --- | --- |
| **West Gorton (Intervention)** | **Pendleton (Control)** |
| *e.g., playground area in park, outside OneStop etc* | *e.g., outside Lidl, outside Clarendon Leisure Centre etc* |
