# Supplementary figures and images for: Long-term impacts of co-designed sustainable park improvements on physical activity and other wellbeing behaviours: a 7-year natural experimental study in a deprived urban area
Source: Int J Behav Nutr Phys Act. 2026 Apr 21;23:60. doi: 10.1186/s12966-026-01918-9 (PMC13237973; doi:10.1186/s12966-026-01918-9)

## Additional file 5. Logic model

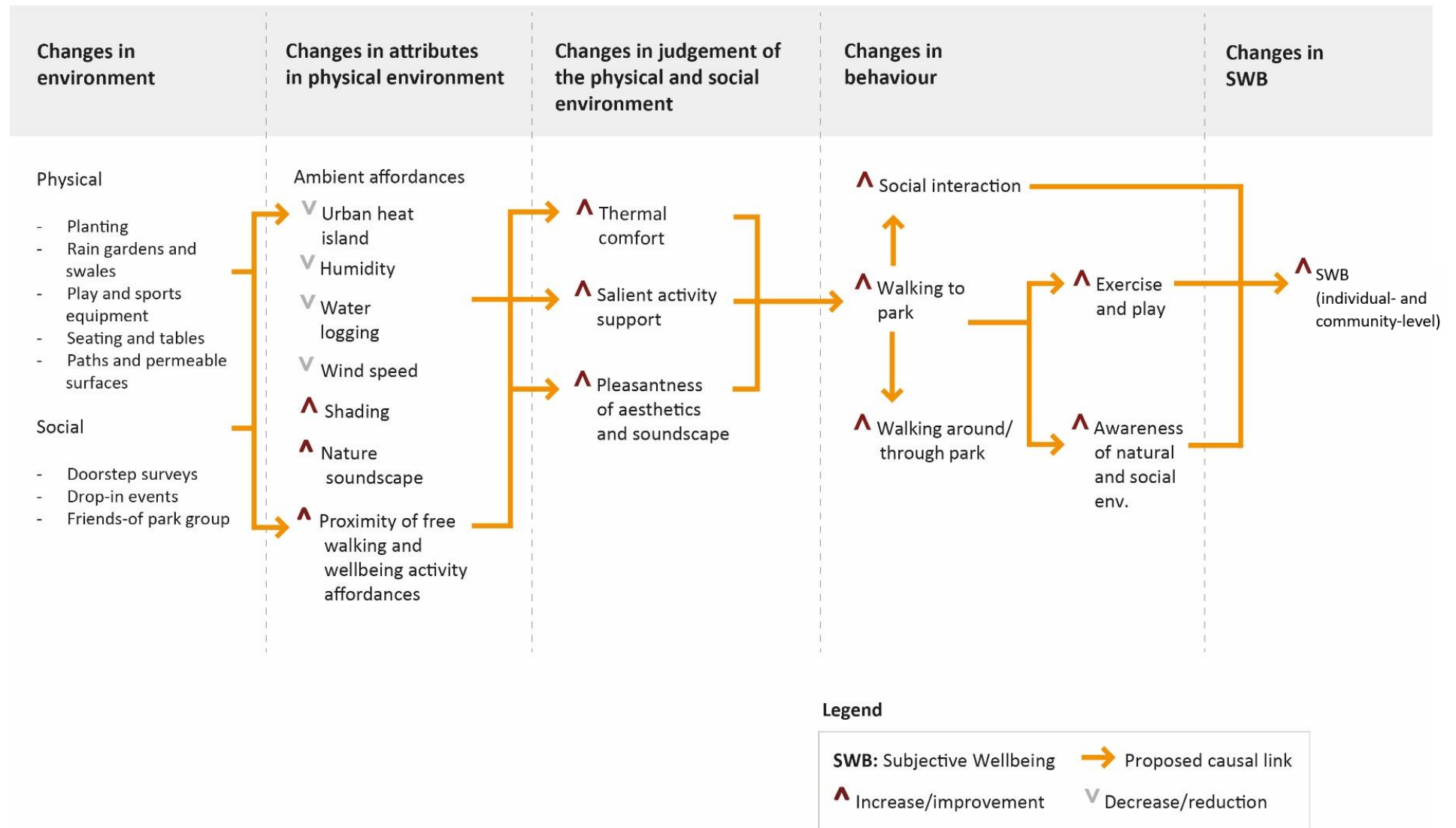

Supplement: Supplementary file 7 — Additional file 7. Logic model. [file 12966_2026_1918_MOESM7_ESM.pdf]
