# Supplementary material for: Long-term impacts of co-designed sustainable park improvements on physical activity and other wellbeing behaviours: a 7-year natural experimental study in a deprived urban area
Source: Int J Behav Nutr Phys Act. 2026 Apr 21;23:60. doi: 10.1186/s12966-026-01918-9 (PMC13237973; doi:10.1186/s12966-026-01918-9)
Supplement: Supplementary file 8 — Additional file 8. Wider neighbourhood trends analysis. [file 12966_2026_1918_MOESM8_ESM.docx]

**Additional file 6.** Wider neighbourhood trends analysis

**Table S6A.** Mixed-effects negative binomial regression results for total number of people using intervention sites and unchanged nearby sites^1^

| **Time point** | **Total number of people**  **(median per observation period)** | | **IRR** | **95% CI** | ***p*-value** | **Robust SE** | **Cluster random effect variance (site)** |
| --- | --- | --- | --- | --- | --- | --- | --- |
|  | **Intervention group** | **Unchanged nearby sites** |  |  |  |  |  |
| Baseline | 470 (29) | 379 (23) | - | - | - | - | - |
| 5 years | 672 (35) | 342 (18) | 1.65 | 1.24-2.20 | 0.001* | 0.24 | 0.03 |
| CI: Confidence interval; IRR: Incidence rate ratio; SE: Standard error  ^1^One observation period from an unchanged nearby site (which was exceptionally busy) was removed from the analysis  Models adjusted for day of week, time of day, and precipitation;  *Statistically significant at p < 0.05 (z-test, two-tailed) | | | | | | | |

**Table S6B.** Sensitivity analysis including the previously removed observation period

| **Time point** | **Total number of people**  **(median per observation period)** | | **IRR** | **95% CI** | ***p*-value** | **Robust SE** | **Cluster random effect variance (site)** |
| --- | --- | --- | --- | --- | --- | --- | --- |
|  | **Intervention group** | **Unchanged nearby sites** |  |  |  |  |  |
| Baseline | 470 (29) | 379 (23) | - | - | - | - | - |
| 5 years | 672 (35) | 592 (19) | 1.04 | 0.45-2.44 | 0.92 | 0.45 | 0.10 |
| CI: Confidence interval; IRR: Incidence rate ratio; SE: Standard error  Models adjusted for day of week, time of day, and precipitation;  *Statistically significant at p < 0.05 (z-test, two-tailed) | | | | | | | |

**Table S6C.** Mixed-effects negative binomial regression results for total number of people using comparison sites and unchanged nearby sites^1^

| **Time point** | **Total number of people**  **(median per observation period)** | | **IRR** | **95% CI** | ***p*-value** | **Robust SE** | **Cluster random effect variance (site)** |
| --- | --- | --- | --- | --- | --- | --- | --- |
|  | **Main comparison group** | **Unchanged nearby sites** |  |  |  |  |  |
| Baseline | 538 (34) | 379 (23) | - | - | - | - | - |
| 5 years | 516 (35) | 342 (18) | 0.99 | 0.73-1.34 | 0.94 | 0.15 | 0.04 |
| CI: Confidence interval; IRR: Incidence rate ratio; SE: Standard error  ^1^One observation period from an unchanged nearby site (which was exceptionally busy) was removed from the analysis  Models adjusted for day of week, time of day, and precipitation;  *Statistically significant at p < 0.05 (z-test, two-tailed) | | | | | | | |

**Table S6D.** Sensitivity analysis including the previously removed observation period

| **Time point** | **Total number of people**  **(median per observation period)** | | **IRR** | **95% CI** | ***p*-value** | **Robust SE** | **Cluster random effect variance (site)** |
| --- | --- | --- | --- | --- | --- | --- | --- |
|  | **Main comparison group** | **Unchanged nearby sites** |  |  |  |  |  |
| Baseline | 538 (34) | 379 (23) | - | - | - | - | - |
| 5 years | 516 (35) | 592 (19) | 0.67 | 0.29-1.57 | 0.36 | 0.29 | 0.12 |
| CI: Confidence interval; IRR: Incidence rate ratio; SE: Standard error  Models adjusted for day of week, time of day, and precipitation;  *Statistically significant at p < 0.05 (z-test, two-tailed) | | | | | | | |
