# Supplementary material for: Long-term impacts of co-designed sustainable park improvements on physical activity and other wellbeing behaviours: a 7-year natural experimental study in a deprived urban area
Source: Int J Behav Nutr Phys Act. 2026 Apr 21;23:60. doi: 10.1186/s12966-026-01918-9 (PMC13237973; doi:10.1186/s12966-026-01918-9)
Supplement: Supplementary file 9 — Additional file 9. Exploratory analyses. [file 12966_2026_1918_MOESM9_ESM.docx]

**Additional file 7.** Exploratory analyses

**Table S7A.** Mixed-effects negative binomial regression results for exploratory outcomes showing effects from baseline to five years

| **MOHAWk demographic characteristic** | **Time point** | **Total counts**  **(median per observation period)** | | **IRR** | **95% CI** | ***p*-value** | **Robust SE** | **Cluster random effect variance (site)** |
| --- | --- | --- | --- | --- | --- | --- | --- | --- |
|  |  | **Intervention group** | **Comparison group** |  |  |  |  |  |
| Non-white | Baseline | 130 (7) | 129 (6) | - | - | - | - |  |
|  | 15 months | 412 (25.5) | 88 (5.5) | 5.01 | 4.34-5.79 | <.0001* | 0.37 | 6.92e-32 |
|  | 5 years | 362 (17) | 213 (11.5) | 1.82 | 1.36-2.43 | <.0001* | 0.27 | 3.43e-32 |
| White | Baseline | 343 (15.5) | 387 (22) | - | - | - | - |  |
|  | 15 months | 421 (23.5) | 235 (14) | 2.30 | 1.43-3.71 | 0.001* | 0.56 | 0.09 |
|  | 5 years | 303 (17.5) | 293 (15) | 1.24 | 0.76-2.00 | 0.39 | 0.30 | 0.07 |
| Young people^1^ | Baseline | 126 (6) | 172 (7) | - | - | - | - | - |
|  | 15 months | 290 (14) | 80 (3) | 5.83 | 1.87-18.17 | 0.002* | 3.38 | 0.33 |
|  | 5 years | 207 (10.5) | 112 (6.5) | 2.04 | 1.62-2.57 | <.0001* | 0.24 | 0.19 |
| Adults | Baseline | 346 (18.5) | 342 (23) | - | - | - | - | - |
|  | 15 months | 542 (38) | 225 (15) | 2.54 | 2.33-2.77 | <.0001* | 0.11 | 0.03 |
|  | 5 years | 442 (27) | 375 (26) | 1.31 | 1.03-1.66 | 0.03* | 0.16 | 0.03 |
| Older adults | Baseline | 21 (1) | 34 (2) | - | - | - | - |  |
|  | 15 months | 21 (1) | 22 (1.5) | N/A^2^ | N/A | N/A | N/A | N/A |
|  | 5 years | 23 (1) | 29 (1) | 1.42 | 0.33-6.07 | 0.64 | 1.05 | 0.21 |
| Females | Baseline | 188 (11) | 233 (13) | - | - | - | - |  |
|  | 15 months | 330 (18.5) | 129 (7.5) | 3.53 | 1.96-6.34 | <.0001* | 1.06 | 0.09 |
|  | 5 years | 257 (12.5) | 212 (14) | 1.57 | 0.99-2.48 | 0.053 | 0.37 | 0.04 |
| Males | Baseline | 295 (17) | 308 (20) | - | - | - | - |  |
|  | 15 months | 515 (31.5) | 193 (12.5) | 3.13 | 2.29-4.28 | <.0001* | 0.50 | 0.01 |
|  | 5 years | 408 (22.5) | 294 (19) | 1.59 | 1.28-1.97 | <.0001* | 0.17 | 0.02 |
| CI: Confidence interval; IRR: Incidence rate ratio; SE: Standard error  Models adjusted for day of week, time of day, and precipitation;  ^1^ Includes infants, children and teenagers  ^2^ Model did not converge  * Statistically significant at p < 0.05 (z-test, two-tailed) | | | | | | | | |
