# Supplementary material for: Long-term impacts of co-designed sustainable park improvements on physical activity and other wellbeing behaviours: a 7-year natural experimental study in a deprived urban area
Source: Int J Behav Nutr Phys Act. 2026 Apr 21;23:60. doi: 10.1186/s12966-026-01918-9 (PMC13237973; doi:10.1186/s12966-026-01918-9)
Supplement: Supplementary file 10 — Additional file 10. Intercept survey participant demographics. [file 12966_2026_1918_MOESM10_ESM.docx]

**Additional file 8.** Intercept survey participant demographics

| **Category** | **Sub-category** | **Time point** | **Intervention group** | **Comparison group** |
| --- | --- | --- | --- | --- |
| Sample size | All adults | Baseline | 101 | 122 |
|  |  | 5 years | 114 | 118 |
| Age group | Adult | Baseline | 89% | 83% |
|  |  | 5 years | 90% | 90% |
|  | Older adult | Baseline | 11% | 17% |
|  |  | 5 years | 10% | 10% |
| Gender | Female | Baseline | 56% | 54% |
|  |  | 5 years | 55% | 42% |
| Ethnic group | Non-white | Baseline | 35% | 19% |
|  |  | 5 years | 59% | 47% |
